# Remote fitness assessment in younger and middle-aged to older adults: a comparison between laboratory- and videoconference-based assessment of selected measures of physical and cognitive fitness

**DOI:** 10.1186/s13102-024-00985-4

**Published:** 2024-09-25

**Authors:** Paula Theobald, Fabian Herold, Thomas Gronwald, Notger G. Müller

**Affiliations:** 1 Research Group Degenerative and Chronic Diseases, Movement, Faculty of Health Sciences Brandenburg, University of Potsdam, Potsdam, 14476 Germany; 2 Institute of Interdisciplinary Exercise Science and Sports Medicine, MSH Medical School Hamburg, Hamburg, 20457 Germany; 3 G-Lab, Faculty of Applied Sport Sciences and Personality, BSP Business and Law School, Berlin, 12247 Germany

**Keywords:** Reliability, eHealth, Cognition, Physical performance, Rural Healthcare

## Abstract

**Background:**

Digital technologies can play an important role in improving the limited accessibility of healthcare services in rural regions (e.g., via remote assessment). However, whether remote fitness assessments (RFA) of selected physical and cognitive fitness parameters are feasible both in younger and older persons and whether they can reproduce laboratory tests needs yet to be established. Thus, this study aimed to address this knowledge gap by investigating the feasibility, and reproducibility of RFA in younger and middle-aged to older adults (MOA).

**Methods:**

A total of 31 younger adults and 32 MOAs participated in this study. At an interval of seven days, laboratory-based and remote assessments (via videoconferencing software) were conducted which included the quantification of the following parameters: (i) measurement of heart rate variability [HRV]; followed by (ii) cognitive testing to examine the level of attention, executive functions (oral Trail Making Test [A and B]), working memory, verbal short-term memory (digit span memory test and word list test (immediate recall)) and episodic memory (word list test (delayed recall)); followed by (iii) physical fitness assessments including performance tests of balance (balance test), functional strength ability of the lower limbs (5-time-sit-to-stand-test) and endurance capacity (3-min step test). Parameters of absolute and relative reliability were determined to assess the reproducibility of the laboratory-based and remote assessments.

**Results:**

The selected physical and cognitive fitness parameters showed moderate to excellent relative reliability (intraclass correlation coefficient [ICC] = 0.52—0.95). The parameters of absolute reliability (Bland–Altman plot and standard error of measurement [SEM]) provide evidence for good reproducibility of HRV parameters and measures of physical fitness, whereas measures of cognitive fitness showed moderate to good reproducibility. On a descriptive level, the absolute and relative reliability of the selected measures of physical and cognitive fitness did not vary as a function of participants’ age.

**Conclusion:**

Our results suggest that RFA of selected measures of physical and cognitive fitness is feasible and reproduces corresponding laboratory results to a moderate to excellent level in both younger adults and MOA. Data showed that the reproducibility of laboratory-based and remote assessments is not influenced by the age of the participants. These findings support the use of digital technologies to improve the accessibility of healthcare services (e.g., in remote areas). However, as the reproducibility varies considerably across the different parameters, further studies are needed to evaluate the effects of an optimised standardisation of the remote assessments and confounding factors.

**Supplementary Information:**

The online version contains supplementary material available at 10.1186/s13102-024-00985-4.

## Introduction

Typically, laboratory assessments are considered the gold standard for testing physical and cognitive fitness both in science and clinical practice because a controlled laboratory-based test environment allows for a high level of standardization, which, in turn, benefits the reliable assessment of specific parameters. However, one disadvantage of a laboratory-based assessment approach is that the participants need to travel to the laboratory or healthcare institution. This can be particularly challenging for younger and older individuals with physical and cognitive disabilities from rural regions [1–3]. Hence, there is a certain risk of selection bias in laboratory-based scientific studies which limits their generalisability because of the under-representation of specific populations (e.g., older adults with cognitive impairment from rural areas) [1, 4].

Beyond scientific applications, this issue is also important with respect to healthcare. Especially for older and/or immobile adults in rural areas, it is often difficult to reach healthcare services such as healthcare specialists or rehabilitation centres [3, 5–8] due to an age- or disease-related decline in life-space mobility (e.g., caused by age-related decline in driving ability) [2, 3, 9, 10].

In this context, new technologies and methods of care (e.g., digital health applications or digital/remote assessments) bear great potential because they can improve (i) the representation of individuals from rural areas in research [1, 11, 12] and (ii) the accessibility of health-related services, which is of high practical relevance for regular healthcare or treatment, and can be used, for example, to complement medical routine assessments [4, 13–15].

Although the application of digital tools is not without limitations (e.g., requiring digital infrastructure and digital skills) [4, 16, 17], several studies [18–22] have shown that digital health applications, for instance, can be used to deliver physical training interventions [19, 23–26] and behavioural therapies in younger and older adults [21, 27]. More specifically, recent systematic reviews on digital training interventions observed significant improvements in various health-related outcome parameters in older adults (e.g., cognitive performance) as compared to the inactive control groups [28, 29].

In addition to online interventions, (remote) assessment of health-related parameters with digital technologies (e.g., videoconference software) has become increasingly popular because of an accelerated digitalization of the public health sector that has been considerably boosted by the COVID-19 pandemic. In several studies, physical and cognitive outcomes were successfully assessed via remote technology [30–33] suggesting that RFA is feasible, valid, and reliable in different cohorts [34–38]. In particular, this research observed that parameters of health-related fitness (e.g., cardiorespiratory fitness determined via step test [39]) and of cognitive performance [40–42] can be reliably evaluated via a remote assessment. Although the above-presented evidence suggests that remote testing is a promising approach to quantify parameters of health-related outcomes in remote settings (e.g., rural areas), a recent systematic review by Heslop and colleagues [16] pointed out that the reviewed studies often did not apply the state-of-the-art and recommended statistical analysis procedures to accurately and robustly compare face-to-face (e.g., in the laboratory) to remote assessments (e.g., via videoconference software) [16]. Thus, the constructive criticism raised by Heslop and colleagues [16] implies that further well-designed studies (e.g., with rigorous statistical analysis of accuracy and precision) are necessary to provide more robust evidence on the accuracy and reproducibility of remote testing. This issue has been addressed in this study through the implementation of a comprehensive set of statistical analysis of reproducibility, utilising procedures such as the analysis of absolute reliability proposed by Bland and Altman [43] missing in several previous studies [16].

In addition, there is still a lack of research on the reproducibility of remotely conducted test batteries (i) that include both physical and cognitive fitness measures and (ii) that are conducted in real-life settings (i.e., at home with personal digital devices). The latter facts support the idea that further investigations on the reproducibility of remote assessments are necessary before they can be unreservedly recommended for practical applications [34, 41, 44].

In this regard, the current study aimed to investigate the feasibility and reproducibility of the assessment of selected physical and cognitive fitness measures in younger adults and MOAs with digital communication technologies such as video conferencing software. In other words, we investigated whether laboratory-based results can be reproduced in a remote setting (i.e., data assessment via videoconference software at the participants’ home). Here, we use the term reproducibility as an umbrella term that includes both, parameters of reliability and parameters of agreement, reporting results on absolute reliability (i.e., via Standard Error of Measurement, and Bland–Altman-Plot) and relative reliability (i.e., via Intraclass Correlation Coefficient) [45, 46]. Based on the current literature [34, 36, 37, 40, 47], we hypothesised that in younger adults and MOA the remote assessment of selected physical and cognitive fitness measures via videoconference software is feasible and can yield to comparable performance outcomes as the laboratory-based testing. In addition, to extend the current knowledge on RFA, we recruited and assessed both younger and middle-aged to older adults (MOA) because available studies on videoconference-based RFA focussed mainly on older adults [36–38] limiting the generalizability of their findings to younger cohorts and thus necessitating further research in this age group. Moreover, in this study, only healthy participants were included to reduce the variability that may arise from short-term changes in disease status which may negatively influence the estimation of reproducibility.

## Method

### Study design

In this study, we investigated the feasibility and reproducibility of physical and cognitive fitness assessments in younger adults and MOA that were conducted in-person in the laboratory and remotely via videoconference software (i.e., Zoom Video Communications®, Inc., CA, USA). Orienting on previous studies [36, 37, 48, 49] the two appointments were separated by an interval of seven days, with the laboratory testing always taking place first (see below for a justification), and scheduled at the same time of day. Comparable to previous studies [37], we selected assessments that required minimal material and space allowing a straightforward implementation in the home of the participants even under limited space conditions. All performance tests were supervised by the same unblinded investigator (P.T.). The study was approved by the local ethics committee of the University of Potsdam (No.40/2022).

#### Participants

A total of 63 subjects (female: *n* = 38, male: *n* = 25) aged between 20 and 35 (younger adults: *n* = 31) and 45 and 75 years (MOA: *n* = 32) were recruited from Potsdam and the surrounding areas. An overview of the participants is provided in Table 1. The recruitment took place via advertisements in newspapers, sports and cultural clubs, posters, and the online recruitment tool of the University of Potsdam between September 2022 to August 2023.

**Table 1:** Overview of the general characteristics of the participants (*N* = 63)

Characteristic	Value		
	*Younger adults (n* =*31)*	*MOA* *(n=32)*	*Total* *(n* *=63)*
**Age (years), mean (± SD)**	25.90 (± 4.37)	61.94 (± 6.23)	44.21 (± 18.93)
**Sex, No**			
** Female**	24 (77.4%)	14 (43.8%)	38 (60.3%)
** Male**	7 (22.6%)	18 (56.3%)	25 (39.7%)
** Body height (cm), mean (± SD)**	170.18 (± 8.87)	174.26 (± 8.60)	172.24 (± 8.90)
** Body weight (kg), mean (± SD)**	65.43 (± 12.74)	77.28 (± 11.60)	71.45 (± 13.47)
** BMI (kg/m2), mean (± SD)**	22.41 (± 2.59)	25.33 (± 2.35)	23.89 (± 2.86)
**Resting blood pressure (mmHg), mean (± SD)**			
** Systolic**	113.87 (± 11.89)	131.53 (± 11.58)	122.84 (± 14.57)
** Diastolic**	75.35 (± 6.97)	85.78 (± 10.83)	80.65 (± 10.48)
** Resting heart rate, mean (± SD)**	72.49 (± 11.49)	69.80 (± 12.34)	71.13 (± 12.17)
**Self-reported diseases, No**			
** None**	27 (87,1%)	15 (46.9%)	24 (66.7%)
** 1 disease**	4 (12.9%)	10 (31.3%)	14 (22.2%)
** 2 diseases**	0	5 (15.6%)	5 (7.9%)
** 3 diseases**	0	2 (6,3%)	2 (3.2%)
**Self-reported medication use, No**			
** None**	27 (87.1%)	11 (34.4%)	38 (60.3%)
** 1 medication**	4 (12.9%)	12 (37.5%)	16 (25.4%)
** 2 medications**	0	7 (21.9%)	7 (11.1%)
** ≥ 3 medications**	0	2 (6.3%)	2 (3.2%)
**Smoker**			
** yes**	4 (12.9%)	4 (12.5%)	8 (12.7%)
** no**	27 (87.1%)	28 (87.5%)	55 (87.3%)
**Alcohol consumption, No**			
** Never (absolute renunciation)**	3 (9.7%)	2 (6.3%)	5 (7.9%)
** ≤ Occasionally (more than 2 times a month)**	18 (58.0%)	7 (21.9%)	25 (39.6%)
** Regularly (several times a week)**	8 (25.8%)	15 (46.9%)	23 (36.5%)
** Daily (more than 200 ml per day)**	2 (6.5%)	7 (21.9%)	9 (14.3%)
** Depression score (PHQ-9), mean (± SD)**	3.90 (± 2.37)	2.13 (± 1.84)	3 (± 2.29)
** Stress score (PSS-10), mean (± SD)**	14.48 (± 4.72)	10.38 (± 4.97)	12.40 (± 5.23)
** Sleep score (PSQI), mean (± SD)**	4.74 (± 2.63)	4.75 (± 2.55)	4.75 (± 2.57)
** Physical activity and sports index (PASQ) (min/week), mean (± SD)**	301.86 (± 221.43)	305.60 (± 232.13)	303.75 (± 225.10)
**School education, No**			
** Lower than High School**	2 (6.4%)	9 (28.1%)	11 (17.5%)
** High School**	29 (93.6%)	23 (71.9%)	52 (82.5%)
**Vocational education, No**			
** Still in education**	8 (25.8%)	0	8 (12.7%)
** Vocational training**	6 (19.4%)	11 (34.3%)	17 (27%)
** University**	17 (54.8%)	21 (65.7%)	38 (60.3%)

Participants were required to have access to a smartphone and computer/tablet with a video function. Exclusion criteria were reported the presence of severe neurological, psychological, orthopaedic, and cardiovascular diseases, as well as acute injuries and acute drug use, pregnancy, and poor German language skills (i.e., writing and language comprehension). To screen for the above-mentioned exclusion criteria, all interested participants underwent an initial screening via videoconference. During the screening the following parameters were assessed: cognitive status via the Minimal Mental State Examination [MMSE]; cut-off: ≥ 27 [50, 51]), risk of falls (Falls Efficiency Scale Short [FES Short]; cut-off: ≤ 13 [52, 53]), depressive symptoms (Patient Health Questionnaire [PHQ]-9; cut-off: < 10 [54]), and risk status for physical exercise-related adverse events (Physical Activity Readiness-Questionnaire [PAR-Q] [55, 56]). In accordance with the principles of the Declaration of Helsinki, participants were provided with verbal and written information about the study, and informed consent was obtained from all participants before the onset of the study.

During the recruitment process for the current study, nine interested adults (three from the younger age group, and six from the MOA group) were excluded after the initial screening because of the presence of a disease or injury, lacking technical requirements, incompatible equipment, and distrust in digital applications (see also Figure S1 in the supplementary material). Of the remaining 63 participants, all were able to complete the laboratory and remote (at home) appointments. Two participants had to reschedule the remote assessment for a week later due to disease and for organisational reasons, leading to the extension of the test–retest interval to 14 days.

### Assessment Procedures

**Fig. 1:**
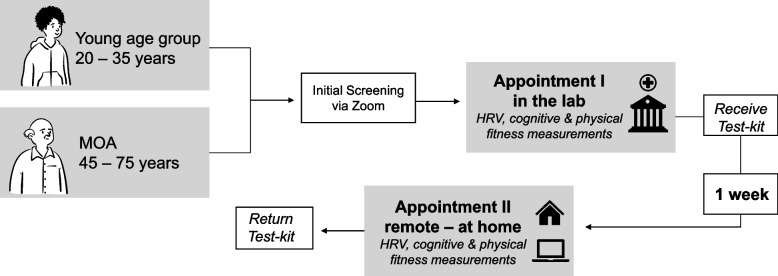
Schematic illustration of the study procedure of the RFA

**Fig. 2:**
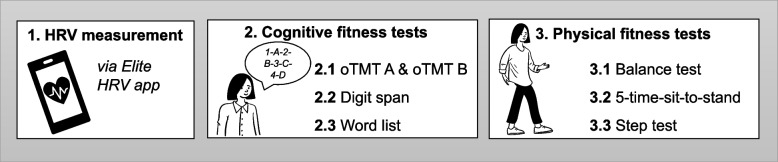
Schematic illustration of the order of the different assessments performed in this study. HRV: heart rate variability; oTMT: oral Trail Making Test

Participants who were identified as eligible in the initial screening underwent two testing appointments. The first examination took place in the laboratory (face-to-face), where the participants started by downloading and configuring the Elite HRV App on their smartphone (Elite HRV Inc., Asheville, NC, USA), which was used to assess heart rate (HR) variability (HRV); see section (i) HRV measurement for a more detailed description). After introducing the app, the first session consisted of (i) the resting-state HRV assessment, followed by (ii) the cognitive fitness tests, and (iii) the physical fitness assessments. Each appointment lasted approximately 60 min and the order of the single assessments was kept constant to avoid effects of order or bias that would arise from physical fitness testing (e.g., the stimulating effect of acute physical exercise on HRV or cognitive performance), respectively. At the end of the first appointment, the participants received a "test-kit" with the materials required for the remote assessments (i.e., containing an HR(V) monitor chest strap, step box, questionnaires, and instructions). One week later the second appointment was conducted remotely via video conferencing software Zoom® at the same time of day to account for the influence of the circadian rhythm on the different parameters of interest (e.g., HRV). Participants were reminded of the upcoming appointments via email and – in case of the second appointment—received the password-protected Zoom®-link. For organisational reasons and based on technical requirements (i.e., informing participants on the handling of the app, Zoom®, and application of the HR(V) monitor chest strap), the home assessment was always performed after the laboratory tests (a schematic illustration of the study procedures is shown in Fig. 1 and 2). In the following, the tests are reported in the same order as they were applied in the study.

(i) HRV recording and analysis:

The resting-state HRV is an easy-to-acquire physiological parameter that can be recorded via miniaturized and portable devices allowing for at-home utilization [57, 58] which provides valuable information about the status of the autonomic nervous system [59] and, in turn, on the health status of the individual [60]. There is considerable evidence suggesting that HRV can be utilized as a biomarker for various diseases (e.g., cardiovascular, cognitive, mental diseases, and frailty) [61–66]. Hence, examining whether HRV can be reliably measured via remote assessments may improve the applicability of this important health measure in the aging population, especially in rural areas. In the current study, we selected the following HRV time domain metrics: log-transformed SDNN (standard deviation of normal-to-normal RR intervals), log-transformed RMSSD (square root of mean squared difference of successive RR intervals) [67–69], and the non-linear metric DFAa1 (short-term scaling exponent alpha1 of detrended fluctuation analysis, window width: 4 ≤ n ≤ 12 beats) as well as the resting HR. In addition to the standard time-related HRV metrics [70], we also decided to include the non-linear parameter DFAa1 because this metric has been shown to be a promising parameter for predicting cardiovascular risk and mortality [71, 72].

The HRV assessments were conducted after downloading and logging into the Elite HRV app by fitting the participants with a chest strap (H10, Polar Electro Oy, Kempele, Finland [73]). A detailed written description of the use of the Elite HRV app was provided to the participants, followed by a detailed oral explanation by the investigator, who supervised the steps from logging into the app to connecting the chest strap to the smartphone (via Bluetooth), both face-to-face and remotely via videoconference. To provide a baseline for a stable HRV signal, participants were familiarised with the measurement situation and were seated comfortably in a quiet environment. They were allowed to start the resting HRV measurement on their own when they felt comfortable to do so. The recording period of the HRV measurement was set to five minutes adhering to the recommendations for short-term HRV recording [74, 75]. The seated participants were asked to breathe freely (i.e., spontaneous breathing patterns without controlling the breathing rate). R-R interval data were transmitted to the Elite HRV app via Bluetooth to the participants’ personal smartphones. The participants were asked to refrain from behaviours that can confound the assessments (e.g., strenuous physical activity 24 h or smoking 3 h before the assessment according to methodological recommendations [70, 74]). After the completion of the HRV measurement, the HRV data (.txt) was downloaded to an external computer and analysed using Kubios HRV 3.5.0 software (Kubios, Ltd., Kuopio, Finland). Pre-processing settings were set to the default values including the RR detrending method which was kept at “smoothness priors” (Lambda = 500). During the post-processing of the HRV data an automatic Kubios artifact algorithm, leading to the correction of 8.15% (± 6.30%) beats in 12 measurements (from a total of 61 measurements), was applied [76, 77]. The analysis period was set to 3 minutes (starting at minute 1:00, and ending at minute 4:00 of the recording period).

(ii) Cognitive fitness measurements:

In accordance with our study aims, we used cognitive tests that can be accomplished with minimal resource requirements (e.g., verbally), making them well-situated to be digitally implemented in low-resource settings. Furthermore, we selected cognitive tests that are well-established instruments in clinical research and diagnostics [41, 78] and that showed moderate to excellent reliability in laboratory and/or remote applications [79–81].

The oral version of the Trail Making Test (TMT A and TMT B) was conducted at the beginning to probe the level of attention and executive functions [82]. In the oral TMT A, participants were asked to count from 1 to 25 as quickly as possible. Oral TMT B differs in that, participants count again, but alternate between numbers and letters (final digit 13). Completion time is the outcome variable, whereby a shorter completion time indicates better performance [83]. Previous studies reported good to moderate reliability of oral TMT assessed via videoconference in healthy older adults (ICC: 0.66 – 0.85) [80]. Afterward, the verbal short-term memory and working memory were quantified via the digit span forward and backward (maximum score: 30 points) [84] and the repetition of a read-out word list (10 words) from the dementia test DemTect (word list immediate recall) (maximum score: 20 points) [81, 85], thereby the tests were appropriate for auditory administration. In both tests, points are allocated for a repeated word or a repeated sequence of digits. A higher score indicates better performance by the participant. In previous studies using videoconference in older adults, the digit span showed moderate reliability (ICC: 0.69 to 0.72) [49]. The reliability of the dementia test DemTect has been reported as excellent (ICC: 0.966) in previous face-to-face studies involving older adults with cognitive impairment [79, 81]. After a five-minute break, in which the balance test was performed, the repetition of the already read-out words from the DemTect (word list delayed recall) was conducted to examine the level of episodic declarative memory (maximum score: 10 points) [81, 85]. At the second examination date in the remote setting, the alternative version of the DemTect subtests was applied to minimize learning effects.

(iii) Physical fitness measurements:

Based on previous research on RFA [16, 37, 39] we selected physical fitness tests that require minimal material (e.g., low-cost tools such as boxes for the step test) and space resources, that can be performed with continuous contact with the investigator, that permit information on three important fitness dimensions (i.e., endurance, strength, balance) in older adults [86], and that are applicable regardless of the age and physical fitness of the participants. Additionally, all selected physical fitness tests are widely used both in research and in clinical settings (e.g., in rehabilitation clinics, geriatric clinics, and sports medicine) [87].

At first, the clinical balance test, which is based on the work of Theisen and Wydra [88], was completed between the last two cognitive fitness tests (i.e., immediate and delayed recall). In brief, three balance tasks were performed with either eyes open or eyes closed for a maximum duration of 15 s each (a more detailed description can be found in the supplementary material). Performance in the balance task was evaluated using a specific scoring system (maximum score: 18 points). Subsequently, the participants completed an established strength and mobility test, namely the 5-time-sit-to-stand-test to determine lower extremity strength [89, 90] and a step test to determine endurance performance (i.e., adapted YMCA-Step-Test [39]). The meta-analysis from Muñoz-Bermejo et al. [90] reported excellent reliability of the 5-time-sit-to-stand-test in laboratory settings (ICC: 0.928–0.937) [90]. Other studies showed that the 3-minutes step test is a valid test for the prediction of the maximal oxygen uptake (VO_2MAX_) in healthy adults [91] as well as showing excellent reliability in a remote setting utilizing videoconferencing for the assessment (ICC: 0.907) [39].

In order to perform the step test remotely, a mobile box (height 27 cm) was used. The appropriate step frequency was calculated using a formula provided by Matthews et al. [39], which was 105 beats per minute provided to the participants via a metronome for the entire duration of the test (i.e., 3 minutes). Individual HR was recorded during the entire step test and after its cessation (for 5 minutes) using the H10 chest strap sensor and the Elite HRV app. The individual VO_2MAX_ was then calculated for each participant using the following formula: VO_2MAX_ [mL/kg/min] = -0.2805 × HR (1 minutes post-exercise) + 76.71 [39]. To ensure an accurate evaluation of the physical fitness assessments and estimation of the participants’ current state, the participants were asked (i) to place their device’s camera in a position allowing the assessor to see their body during the entire remote setting, and (ii) to not turn off the sound during the physical fitness assessments.

#### Other measurements

Before each appointment (i.e., laboratory and remote), participants completed the Brief Sport Recovery and Stress Scale [SRSS] [92] to identify possible changes in recovery and stress between conditions. After each appointment, with a self-administered questionnaire (see questionnaires I and II in the supplementary material for details) participants gave their subjective view about how they had perceived the assessments (using a 5-point Likert scale).

In addition to the assessments and the two above-mentioned questionnaires, we also collected anthropometric data (body height, body weight, resting blood pressure) of the participants and some other health-related and demographic data (i.e., participants’ demographic and socioeconomic background, nutritional patterns, alcohol and nicotine consumption, diseases, and medication regimens) using a self-administered questionnaire during the laboratory-based assessment (see questionnaire III in the supplementary material for details).

To obtain further information about the lifestyle of the participants, the following questionnaires were supplied with the test-kit and answered by the participants at their homes: the Perceived Stress Scale [PSS], which measures stress levels over the past four weeks [93]; the Physical Activity and Sports Questionnaire [PASQ], which evaluates physical activity retrospectively over the past 4 weeks [94]; and the Pittsburgh Sleep Quality Index [PSQI], which provides a sleep index over the past 4 weeks [95]. 

### Statistics

Data analysis was conducted using SPSS Statistics software (IBM SPSS Statistics for Windows, Version 28.0.1.1. Armonk, NY: IBM Corp.) and the significance level was set to α ≤ 0.05. The descriptive statistics are reported as mean values (M) and standard deviations (SD). The statistical analysis of reproducibility was performed as follows. First, the data were tested for normal distribution (using Shapiro–Wilk test) and homoscedasticity. Subsequently, the presence or absence of systematic errors was determined using paired t-tests [45, 96, 97]. The relative reliability was calculated using an intraclass correlation coefficient (ICC) with a 2-way mixed model (absolute agreement type; ICC 3.1) [46]. According to Koo and Li [46], we rated the ICC as follows: poor (< 0.5), moderate (0.5- 0.75), good (0.75 -0.9), and excellent (≥ 0.9) relative reliability. As measures of absolute reliability, we determined, the standard error of the measurements (SEM) and created Bland–Altman plots with 95% limits of agreement (LoA) (mean difference ± 1.96 SD of differences [97, 98]). The SEM was calculated using the following formula: $SEM=SD\sqrt{1-ICC}$ [45, 46, 98]. A SEM smaller than 10% of the mean laboratory and remote score was considered to indicate acceptable absolute reliability [36, 38, 97]. The interpretation of effect sizes is based on Cohen’s for small effects (*d* ≥ 0.2), moderate effects (*d* ≥ 0.5), and large effects (*d* > 0.8) [99].

## Results

### Demographics and characteristics

Our sample consisted of younger adults (*n* = 31) aged between 20–35 years (25.90 ± 4.37 years) and MOA (*n* = 32) aged between 45–75 years (61.94 ± 6.23 years). The sample of younger adults were predominantly healthy women with a high level of education and a low number of participants who smoked (12.9%) or drank alcohol daily (6.5%), while our sample of MOA consisted of slightly more male participants (56.3%) who had received a high level of education and a smaller number of smokers (12.5%). However, on a descriptive level, alcohol consumption was slightly higher in the MOA compared to the younger participants (old cohort: 46.9% regular alcohol consumption, 21.9% daily alcohol consumption). A higher number of participants in the sample of MOA reported having a disease and taking medication compared to the younger adults (see Table 1). Overall, our sample of 63 participants can be described as predominantly healthy (66.7% without disease), female (60.3%), and with a high level of education (82.5% high school graduation, 60.3% university diploma).

### Reproducibility results – younger adults

Except for missing values for individual parameters of single participants, all 31 younger participants were included in the reproducibility analysis. Table 2 reports the results of the ICC (with a 95% confidence interval), as well as the SEM of the specific parameters of the assessment. Table 3 shows the limits of agreement with the mean of the difference between remote- and laboratory-based settings. In addition, the Brief Sport Recovery and Stress Scale conducted at the beginning of the two assessments indicated no statistically significant differences between the perception of stress and recovery as well as sleep behaviour and sleep quality between the two study settings (see Table S1 in the supplementary material for details).

**Table 2:** Overview of the intraclass correlation coefficient (ICC) and the standard error of measurement (SEM) for the comparison between the laboratory and remote assessments in the sample of younger adults

Measure	Laboratory Mean (SD)	Remote Mean (SD)	ICC (95% CI)	*p*-value	SEM (%)^a^
**lnRMSSD (ms) [*n*** **= 30]**	3.56 (0.52)	3.67 (0.56)	0.66 (0.40–0.81)	< 0.001	0.32 (8.8%)
**lnSDNN (ms) [*n*** **= 30]**	3.75 (0.43)	3.83 (0.44)	0.62 (0.34–0.80)	< 0.001	0.27 (7.0%)
**DFAa1 [*n*** **= 30]**	1.12 (0.30)	1.10 (0.31)	0.65 (0.39–0.82)	< 0.001	0.18 (16.2%)
**Resting HR (bpm) [*n*** **= 30]**	73.22 (11.90)	71.76 (12.14)	0.77 (0.58–0.88)	< 0.001	5.51 (7.6%)
**Step test (VO**_**2MAX**_** [mL/kg/min]) [*n*= 28]**	46.09 (5.97)	46.79 (6.03)	0.91 (0.82–0.96)	< 0.001	1.75 (3.8%)
**Oral TMT A (sec) [*n*** **= 31]**	9.56 (4.24)	10.20 (4.24)	0.93 (0.85–0.97)	< 0.001	1.10 (11.1%)
**Oral TMT B (sec) [*n*** **= 31]**	32.99 (16.91)	29.44 (17.13)	0.68 (0.44–0.83)	< 0.001	9.56 (30.6%)
**Digit span (points) [*n*** **= 31]**	19.03 (3.92)	18.61 (4.363)	0.75 (0.55–0.87)	< 0.001	2.05 (10.9%)
**Word list immediate recall (points) [*n*** **= 31]**	15.68 (2.78)	16.26 (2.57)	0.52 (0.21–0.73)	< 0.001	1.86 (24.3%)
**Word list delayed recall (points) [*n* = 30]**	7.73 (1.80)	7.63 (1.69)	0.60 (0.30–0.79)	< 0.001	1.09 (6.8%)
**Balance (points) [*n*= 31]**	16.29 (1.44)	16.03 (1.30)	0.54 (0.24–0.75)	< 0.001	0.93 (5.7%)
**5-time-sit-to-stand (sec) [*n*= 31]**	7.73 (2.02)	8.25 (2.25)	0.89 (0.71–0.95)	< 0.001	0.72 (9.0%)

*Bpm* beats per minute, *CI* Confidence interval, *DFAa1* short-term scaling exponent alpha1 of detrended fluctuation analysis, *HR* heart rate, *ICC* Intra-class correlation coefficient, *ln* natural log-transformed, *RMSSD* square root of mean squared difference of successive RR intervals, *SDNN* standard deviation of normal-to-normal RR intervals, *SD* standard deviation, *TMT* Trail Making Test, *VO*_*2MAX*_ maximum oxygen uptake

^a^SEM expressed as percentage of the mean laboratory and remote score

**Table 3:** Overview of the Bias (Mean Difference) and Limits of Agreement (LoA) for the comparison between the laboratory and remote assessments in the sample of younger adults

Measure	Mean Difference (SD)	**Limits of Agreement**	
		Lower Limit	Upper Limit
**lnRMSSD (ms) [*n*** **= 30]**	-0.11 (0.45)	-0.99	0.76
**lnSDNN (ms) [*n*** **= 30]**	-0.09 (0.38)	-0.82	0.65
**DFAa1 [*n*** **= 30]**	-0.03 (0.26)	-0.48	0.53
**Resting HR (bpm) [*n*** **= 30]**	1.45 (8.10)	-14.42	17.33
**Step test (VO**_**2MAX**_ **[mL/kg/min]) [*n*** **= 28]**	-0.70 (2.42)	-5.44	4.05
**Oral TMT A (sec) [*n*** **= 31]**	-0.63 (1.48)	-3.54	2.27
**Oral TMT B (sec) [*n*** **= 31]**	3.65 (1.61)	-23.02	30.32
**Digit span (points) [*n*** **= 31]**	0.50 (2.94)	-5.27	6.27
**Word list immediate recall (points) [*n*** **= 31]**	-0.50 (2.63)	-5.66	4.66
**Word list delayed recall (points) [*n*** **= 30]**	0.10 (1.58)	-3.00	2.27
**Balance (points) [*n*** **= 31]**	0.26 (1.38)	-2.43	3.00
**5-time-sit-to-stand (sec) [*n*** **= 31]**	-0.53 (0.94)	-2.38	1.32

*Bpm* beats per minute, *DFAa1* short-term scaling exponent alpha1 of detrended fluctuation analysis, *HR* heart rate, *ln* natural log-transformed, *RMSSD* square root of mean squared difference of successive RR intervals, *SDNN* standard deviation of normal-to-normal RR intervals, *SD* standard deviation, *TMT* Trail Making Test, *VO*_2MAX_ maximum oxygen uptake

**Fig. 3:**
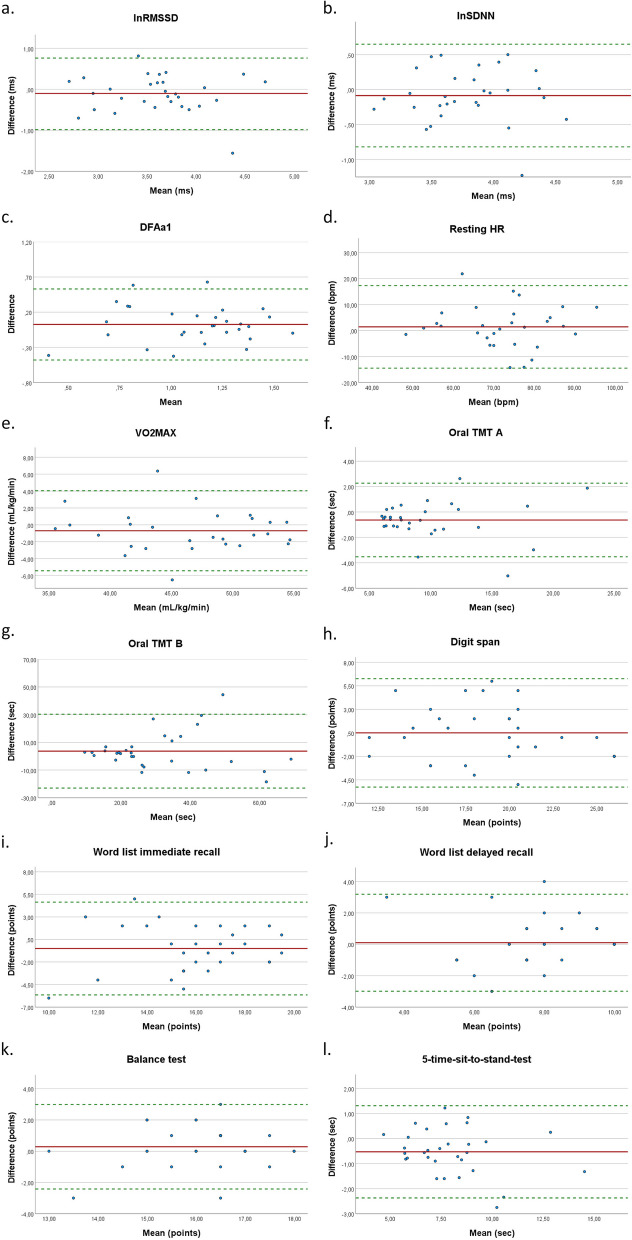
Bland–Altman plots visualizing the agreement between laboratory-based and remote assessments in the sample of younger adults: (**a**) lnRMSSD [natural log-transformed square root of mean squared difference of successive RR intervals]; (**b**) lnSDNN [natural log-transformed standard deviation of normal-to-normal RR intervals], (**c**) DFAa1 [short-term scaling exponent alpha1 of detrended fluctuation analysis]; (**d**) Resting HR [heart rate]; (**e**) VO_2MAX_ [maximum oxygen uptake]; (**f**) Oral TMT A [Trail Making Test A]; (**g**) Oral TMT B [Trail Making Test B]; (**h**) Digit span; (**i**) Word list immediate recall; (**j**) Wordlist delayed recall; (**k**) Balance test; (**l**) 5**-**time-sit-to-stand-test

(i) HRV measurements – younger adults:

In the sample of younger adults, no significant differences were observed for all HRV parameters (lnRMSSD, lnSDNN, DFAa1, HR) between the laboratory and remote measurements (see Table S1 in the supplementary material for details). The results of the relative reliability analysis, determined by the ICC (together with the 95% confidence interval), showed moderate (ICC: lnRMSSD = 0.66; lnSDNN = 0.62; DFAa1 = 0.65, see Table 2) to good (ICC: resting HR = 0.77) reliability. The results of the absolute reliability, described by the SEM, indicated acceptable reliability for the parameters lnSDNN (7.0%), lnRMSSD (8.8%), and resting HR (7.6%), whereas the parameter DFAa1 displayed higher values (SEM 16.2%). Figure 3a-d illustrates the results of the Bland–Altman plot (with the limits of agreement). The graph shows good agreement between the two assessment settings for all three HRV parameters and resting HR, only a few observations fall outside the limits of agreement.

(ii) Cognitive fitness measurements – younger adults:

Reproducibility varied between the different cognitive tests. All tests, except TMT A, indicated no significant differences between the remote and laboratory-based measurements (see Table S1 in the supplementary material for details). The assessments of word list immediate recall and word list delayed recall demonstrated moderate relative reliability in the sample of younger adults (ICC: word list = 0.52; word list recall = 0.60). The digit span and TMT B tests showed good relative reliability (ICC: digit Span = 0.75; TMT B = 0.68) and the TMT A indicated excellent reliability (ICC: 0.93), but showed a systematic error indicated by a statistically significant t-test.

The absolute reliability was acceptable only for the word list recall assessment (SEM = 6.8%), whereas the results for the TMT A (11.1%) and digit span (10.9%) were slightly > 10%. As shown in Table 2 and Fig. 3, the word list and TMT B tests showed a rather poor absolute reliability based on the analysis of the SEM. However, the Bland–Altman plots showed a good agreement between laboratory-based and remote assessments of the cognitive fitness measures, with only a few outliers, whereby the LoA of TMT B displayed a very large interval (LoA: -23.02 to 30.32).

(iii) Physical fitness measurements – younger adults:

The results of the physical fitness assessments varied considerably between the different tests. The comparison of the step test and the balance test revealed no statistically significant differences between the laboratory-based and remote assessments, but we noticed a statistically significant difference between both settings for the 5-time-sit-to-stand-test (*p* = 0.003). The relative reliability analyses showed excellent values for the step test (ICC: 0.91), good values for the 5-time-sit-to-stand-test (ICC: 0.89, but systematic error indicated by a statistically significant t-test – details in the supplementary material), and moderate results for the balance test (ICC: 0.54). Table 2 provides a more detailed overview of the indices used to assess absolute reliability. The SEM was acceptable for all physical fitness parameters, and the Bland–Altman-plot (see Fig. 3e,k,l) showed good absolute reliability (i.e., indicated by a small bias and close LoA) between the laboratory-based and remote tests, with only a few outliers.

### Reproducibility results – MOA

In the reproducibility analysis, the data of all 32 MOA were included, except for missing values for individual parameters of single participants (see also section feasibility analyses). Furthermore, no significant difference was found in our sample of MOA between the study settings in the perception of stress and recovery, sleep quality, and sleep duration (see Table S2 in the supplementary material for details).

(i) HRV measurements – MOA:

The detailed results of the MOA are shown in Table 4. We observed no statistically significant differences in the HRV parameters between the laboratory-based and remote assessments (see Table S2 in the supplementary material for details). The analysis of the relative reliability, operationalized by the ICC, indicated a moderate to good relative reliability (ICC: lnSDNN = 0.80, lnRMSSD = 0.73, DFAa1 = 0.53, resting HR = 0.84).

**Fig. 4:**
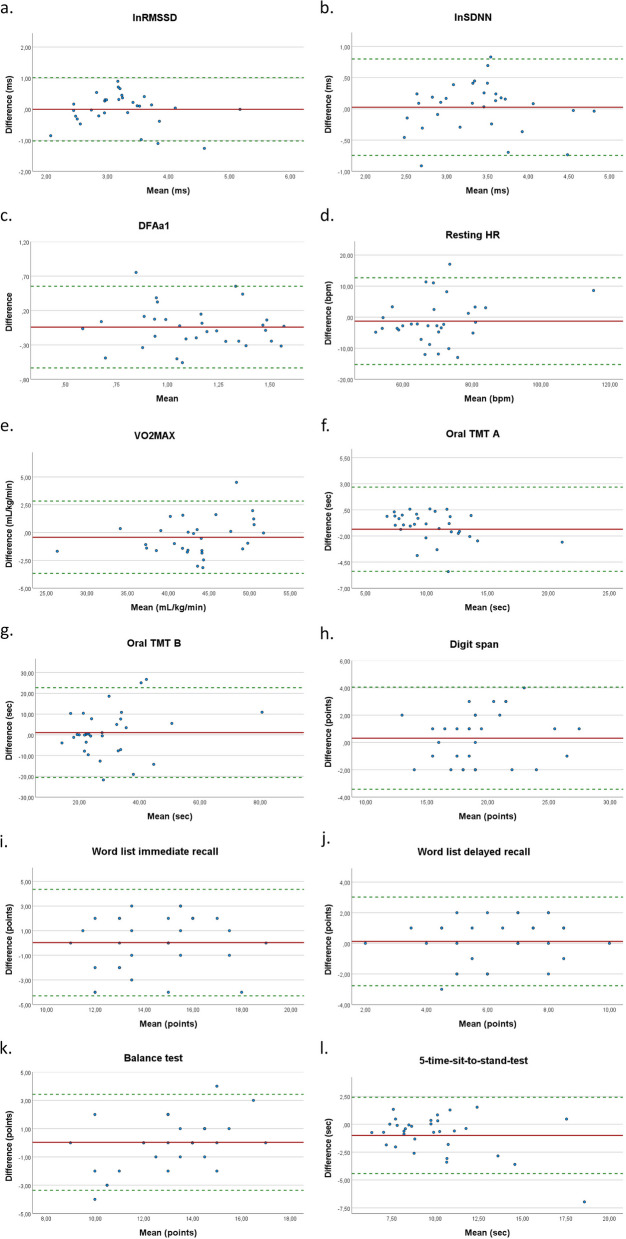
Bland–Altman plots visualizing the agreement between laboratory-based and remote assessments in the sample of MOA: (**a**) lnRMSSD [natural log-transformed square root of mean squared difference of successive RR intervals]; (**b**) lnSDNN [natural log-transformed standard deviation of normal-to-normal RR intervals], (**c**) DFAa1 [short-term scaling exponent alpha1 of detrended fluctuation analysis]; (**d**) Resting HR [heart rate]; (**e**) VO_2MAX_ [maximum oxygen uptake]; (**f**) Oral TMT A [Trail Making Test A]; (**g**) Oral TMT B [Trail Making Test B]; (**h**) Digit span; (**i**) Word list immediate recall; (**j**) Wordlist delayed recall; (**k**) Balance test; (**l**) 5-time-sit-to-stand-test

Regarding absolute reliability, the parameter lnSDNN and resting HR displayed acceptable results with a SEM of 8.1% and 7.1%, whereas the SEM of lnRMSSD (11.2%) and DFAa1 (SEM 18.6%) showed values > 10%. The Bland–Altman plots are shown in Fig. 4a-d, and the corresponding LoA are provided in Table 5. The Bland–Altman plots of the three analysed HRV parameters and resting HR indicated a good agreement between laboratory-based and remote assessments, there are only a few observations that fall outside the limits of agreement.

**Table 4:** Overview of the intraclass correlation coefficient (ICC) and the standard error of measurement (SEM) for the comparison between the laboratory and remote assessments in the sample of MOA

**Measure**	**Laboratory Mean (SD)**	**Remote Mean (SD)**	**ICC (95% CI)**	***p*-value**	**SEM (%)**^a^
**lnRMSSD (ms)[*n*=31]**	3.23 (0.67)	3.24 (0.76)	0.73 (0.51-0.86)	<0.001	0.36 (11.2%)
**lnSDNN (ms) [*n*=31]**	3.39 (0.62)	3.37 (0.63)	0.81 (0.64-0.90)	<0.001	0.27 (8.1%)
**DFAa1 [*n*=31]**	1.12 (0.30)	1.16 (0.32)	0.53 (0.22-0.74)	<0.001	0.21 (18.6%)
**Resting HR (bpm) [*n*=31]**	69.17 (13.28)	70.44 (11.51)	0.84 (0.69-0.92)	<0.001	4.94 (7.1%)
**Step test (VO**_2MAX_ **[mL/kg/min])[*n*=30]**	43.11 (5.76)	43.54 (5.27)	0.95 (0.91-0.98)	<0.001	1.18 (2.7%)
**Oral TMT A (sec) [*n*=32]**	10.11 (2.80)	11.46 (3.99)	0.77 (0.44-0.87)	<0.001	1.68 (15.6%)
**Oral TMT B (sec) [*n*=32]**	30.38 (14.93)	29.30 (12.58)	0.69 (0.45-0.83)	<0.001	7.69 (25.8%)
**Digit span (points) [*n*=32]**	19.50 (3.62)	19.19 (3.35)	0.85 (0.72-0.92)	<0.001	1.34 (6.9%)
**Word list immediate recall (points) [*n*=32]**	14.50 (2.48)	14.47 (2.17)	0.56 (0.26-0.76)	<0.001	1.53 (10.6%)
**Word list delayed recall (points) [*n*=32]**	6.38 (2.21)	6.25 (1.78)	0.70 (0.47-0.84)	<0.001	1.03 (16.2%)
**Balance (points) [*n*=32]**	13.19 (2.55)	13.19 (1.75)	0.67 (0.42-0.82)	<0.001	1.25 (9.5%)
**5-time-sit-to-stand (sec) [*n*=32]**	9.58 (2.59)	10.49 (3.31)	0.80 (0.57-0.90)	<0.001	1.35 (13.5%)

*Bpm* beats per minute, *CI* Confidence interval, *DFAa1* short-term scaling exponent alpha1 of detrended fluctuation analysis, *HR* heart rate, *ICC* Intra-class correlation coefficient, *ln* natural log-transformed, *RMSSD* square root of mean squared difference of successive RR intervals, *SDNN* standard deviation of normal-to-normal RR intervals, *SD* standard deviation, *TMT* Trail Making Test, *VO*_2MAX_ maximum oxygen uptake

^a^SEM expressed as percentage of the mean laboratory and remote score

(ii) Cognitive fitness measurements – MOA:

Except for oral TMT A, the cognitive fitness assessments indicated no statistically significant differences between laboratory-based and remote assessments (see Table S2 in the supplementary material for details). Comparable to our sample of younger adults, the results on reproducibility differed between the specific cognitive fitness tests. The word list immediate recall and oral TMT B demonstrated moderate relative reliability (ICC: word list = 0.56; TMT B = 0.69), the word list delayed recall and oral TMT A showed good relative reliability (ICC: word list delayed recall = 0.70; TMT A = 0.77), and the digit span test achieved excellent relative reliability (ICC: 0.85). The digit span assessment was the only cognitive parameter that achieved acceptable absolute reliability indicated by an SEM of 6.9%. However, the other parameters showed moderate to poor values for absolute reliability (see Table 4 for SEM). In general, the Bland–Altman plots, as shown in Fig. 4f**-**j, hint towards good absolute reliability (agreement) between the assessment settings, whereby the LoA of oral TMT B (-20.56 to 22.71) can be considered as very large.

(iii) Physical fitness measurements – MOA:

Except for the 5-time-sit-to-stand-test, no significant differences were found between the laboratory-based and remote assessments of the physical fitness measurements (see Table S2 in the supplementary material for details). The results of the relative reliability analysis showed moderate relative reliability for the balance test (ICC: 0.67), good relative reliability for the 5-time-sit-to-stand-test (ICC: 0.80, but systematic error indicated by a statistically significant t-test), and excellent reliability for the step test (ICC: 0.95). The balance test and the step test also demonstrated acceptable absolute reliability measures, whereas the 5-time-sit-to-stand-test showed a value > 10% (SEM: 13.5%). Based on the Bland–Altman plots, in our sample of MOA, good absolute reliability (agreement) can be assumed for the various physical fitness parameters between the laboratory-based and remote assessments (see Fig. 4e,k,l).

**Table 5:** Overview of the Bias (Mean Difference) and the Limits of Agreement (LoA) for the comparison between the laboratory and remote assessments in the sample of MOA

**Measure**	**Mean Difference (SD)**	**Limits of Agreement**	
		**Lower Limit **	**Upper Limit **
**lnRMSSD (ms) [*n*=31]**	-0.01 (0.52)	-1.02	1.01
**lnSDNN (ms) [*n*=31]**	0.03 (0.39)	-0.75	0.80
**DFAa1 [*n*=31]**	-0.04 (0.30)	-0.64	0.55
**Resting HR (bpm) [*n*=30]**	-1.27 (7.12)	-15.23	12.69
**Step test (VO**_2MAX_ **[mL/kg/min]) [*n*=30]**	-0.42 (1.66)	-3.68	2.83
**Oral TMT A (sec) [*n*=32]**	-1.35 (2.06)	-5.38	2.68
**Oral TMT B (sec) [*n*=32]**	1.08 (11.04)	-20.56	22.71
**Digit span (points) [*n*=32]**	0.31 (1.91)	-3.43	4.05
**Word list immediate recall (points) [*n*=32]**	0.03 (2.21)	-4.29	4.36
**Word list delayed recall (points) [*n*=32]**	0.13 (1.48)	-2.77	3.02
**Balance (points) [*n*=32]**	0.03 (1.73)	-3.36	3.43
**5-time-sit-to-stand (sec) [*n*=32]**	-1.00 (1.74)	-4.42	2.42

*Bpm* beats per minute, *DFAa1* short-term scaling exponent alpha1 of detrended fluctuation analysis, *HR* heart rate, *ln* natural log-transformed, *RMSSD* square root of mean squared difference of successive RR intervals, *SDNN* standard deviation of normal-to-normal RR intervals, *SD* standard deviation, *TMT* Trail Making Test, *VO*_2MAX_ maximum oxygen uptake

### Comparison between younger adults and MOA

On a descriptive level, the results of the younger adults and the MOA were similar and the reproducibility of our selected measures of physical and cognitive fitness did not vary with age.

More specifically, for the HR and step test, we noticed in both age groups good to excellent relative reliability (ICC: 0.77—0.95) and acceptable absolute reliability (SEM: 2.7%—7.6%). Only for the oral TMT A and the 5-time sit-to-stand test, a significant difference was observed in both cohorts between laboratory and remote-based results, indicating a systematic error in both tests. However, this observation was independent of age. Likewise, the age group did not influence the remaining parameters of HRV, cognitive, and physical fitness, no clear trends concerning the influence of the age group were observed (see Tables 2 and 4 for more details).

### Feasibility analyses

In this study, all of the included participants (*n* = 63) had the technical resources (i.e., internet access, smartphone, computer with video function) to conduct the remote assessments although three MOA participants (*n* = 3) asked their families for support in operating and setting up the technical devices (using the Zoom® software and the computer). In three cases, HR data of the step test (using the Elite HRV app) was lost because of technical or manual error (e.g., inadequate internet connection or an incorrect operation of the app by the participants). Due to a missing Bluetooth connection between the smartphone of the participant and the HR(V) monitor, no HRV and step test data were recorded for the two participants (see details in the supplementary material).

Overall, the participants were coping successfully with the technical requirements at home guided by videoconference. This observation is buttressed by the data from the self-administered questionnaire on the feasibility of remote assessments since no statistically significant difference was found between the participants’ subjective perception of the feasibility in the laboratory and the remote setting. In particular, only 4 of the 63 participants (6.4%), two of the MOA, and two of the younger adults indicated that they think that the completed assessments via videoconference are not feasible for application in practical settings. No study-related adverse events were observed or reported during the entire study period.

## Discussion

The current study investigated in healthy younger adults and MOA the feasibility and the reproducibility of selected measures of physical and cognitive fitness that were captured in-person in the laboratory and one week later remotely via video conferencing software. The key findings of the current study are that both in healthy younger adults and MOA, the remote-based assessment is feasible and shows, in general, moderate to good reproducibility. In the following, we will discuss the findings of our study in more detail.

### Reproducibility of HRV measurements

HRV is an important biomarker for the status of the autonomic nervous system in a variety of diseases [59, 64, 100, 101] highlighting the importance of HRV parameters in clinical and research settings. In general, we observed a moderate to good reproducibility of the HRV parameters when comparing the data of the laboratory to remote assessments (e.g., ICC: 0.53—0.84; SEM: 7.0%—18.6%, for more details, see Tables 2 and 4). However, this observation should be interpreted with caution as the data (RMSSD, SDNN) were log-transformed (and not back-transformed), which might lead to an overestimation of the reproducibility [67]. To the best of our knowledge, we provide the first evidence that HRV parameters can be reliably recorded at the homes of the participants. In general, our observations are consistent with the findings of other laboratory-based studies. For instance, in the study conducted by Uhlig et al. [67], in which a large sample of 120 students aged between 18 and 39 years was measured 5 times during a time interval of 11 months. The indices of relative reliability (e.g., ICC of 0.46 – 0.76) and absolute reliability (e.g., SEM: lnRMSSD = 0.41, lnSDNN = 0.29) were comparable with the results of our sample of younger adults, although the indices of absolute reliability pointed towards a high level of random variability. In addition, the findings of another study by Ashaie et al. [102], who recruited a sample of older participants with aphasia and stroke with a comparable average age (55.76 ± 11.62 years) to our sample of MOA (61.94 ± 6.23 years) mirrors the results of the lnSDNN, lnRMSSD, and DFAa1 parameters of our analysis, despite differences in the experimental design (e.g., one-month interval between two measurements, laboratory-only study, diseased sample) [102]. In contrast, the resting HR results in our study showed a higher relative reliability as compared to the study by Ashaie et al. (ICC = 0.84 vs. 0.45 [102]). Nevertheless, the indices of absolute reliability are relatively equivalent, although our results were slightly better (SEM: DFAa1 = 0.21 vs 0.26 [102]; HR = 4.94 bpm vs. 6.28 bpm [102]). In this regard, there is evidence that intra-individual variability in resting HR can fluctuate between 3 and 10 beats per minute [103], and is influenced by, for instance, diet and physical exercise, suggesting that an SEM of 5.51 bpm (younger sample) and 4.94 bpm (MOA) is within the physiological variability range of a healthy sample. Our finding that resting HR is comparable between remote and laboratory assessment is encouraging because several studies [104, 105] showed that resting HR is an important predictor of mortality and sudden cardiac death. Thus, remote recording of resting HR has the potential to become a promising tool for healthcare services in rural areas.

A study by Hoffmann et al. [69], in which in a laboratory setting an identical test–retest interval was used (i.e., seven days), reported somewhat higher ICCs for HR, lnRMSSD, DFAa1 (ICC: HR = 0.97, lnRMSSD = 0.98, DFAa1 = 0.77). Such an observation is perhaps related to the fact that laboratory-based assessments allow for a higher level of standardisation and that a small sample of elite male athletes was recruited (i.e., no confounding factors such as the female menstrual cycle [106]) which somewhat alters the interindividual variability but also limits the generalisability of their findings [69]. In addition, different analysis settings of HRV measures could lead to slightly different outcomes [74].

On a descriptive level, the relative reliability of the HRV indices in our sample of MOA is only marginally different from that of our younger adults (ICC: MOA sample 0.56–0.84 vs. younger sample 0.62–0.77), but the parameters of absolute reliability are slightly better in younger adults as compared to the MOA (SEM: MOA 7.1%—18.6% vs. younger sample 7.6%—16.2%). Intuitively, one may assume that the above-mentioned marginal difference in HRV indices is perhaps related to the usage of digital devices for the RFA being more stressful for the MOA. However, the recordings of the Brief Sport Recovery and Stress Scale [92] did not show any significant differences between laboratory-based and remote assessments in both young and MOA samples so that the observed marginal lower absolute reliability in older adults may not be attributable to stress. Nevertheless, the digital and unfamiliar assessment situation may contribute, at least partly, to the poorer readings of absolute reliability because they might introduce some interindividual variability in the data of MOA leading to poorer absolute reliability [101, 107].

In this context, it is also important to consider the following points. Firstly, HRV as a physiological parameter is not an optimal time-stable measure because of high intraindividual variations and influencing factors. HRV is prone to the influence of several factors such as physical activity, stress, sleep, menstrual cycle phases, emotions, mood, and breathing patterns [60, 67, 106, 107], all of which can influence reproducibility. To improve the reproducibility of remotely recorded HRV (i.e., at the homes of the participants), we suggest that future studies should consider to increase the number of HRV measurements and aim for a higher standardisation of the assessments [108]. Secondly, to the best of our knowledge, we were the first who investigated the reproducibility of short-term HRV measurements in different test settings, namely laboratory-based versus remote-based assessments utilizing videoconference software. Future research in home-based settings is required to better translate scientific evidence into practice.

### Reproducibility of cognitive fitness measurements

Overall, we observed a considerable variance concerning the reproducibility among the different cognitive tests being applied in the current study so that it is too premature to provide clear recommendations on their applicability for remote videoconference-based assessments in research and practical settings. For instance, in our samples of younger adults and MOA the digit span test showed an acceptable absolute and at least a good relative reliability. This observation somewhat contrasts the results of Munro Cullum et al. [109], who observed in older adults only moderate relative reliability for the digit span test (ICC: 0.545 – 0.590), even though they provided evidence that other cognitive tests (e.g. Mental State Examination, Hopkins Verbal Learning Test-Revised) can be reliably conducted via video conferencing (i.e., retest on the same day, with an average of 21 minutes between the sessions) [109]. In addition, Fox-Fuller et al. [40], who assessed in adults (i.e., aged between 18 and 75) the digit span remotely via videoconferencing at a test–retest interval of 4 to 6 months, reported somewhat lower relative reliability (ICC: 0.61–0.66) [40] as compared to our study. Taken together, the current results suggest that within a relatively short test–retest interval the remote digit span test is highly reliable, whereas other studies (e.g., [40]) imply that the relative reliability can be influenced by the test–retest interval.

Regarding the oral TMT, it is important to consider that in both age groups the time to complete the oral TMT A was statistically shorter in the laboratory as compared to the remote assessment, which hints towards a systematic error that partly may be related to delays in video transmission. These aspects lead to the fact that the results should still be approached with some caution. Nevertheless, the analysis of reproducibility for the oral TMT A showed good to excellent relative reliability and moderate absolute reliability. The oral TMT B shows moderate relative reliability in our sample of younger adults and MOA, which are slightly lower values than in the study by Wadsworth et al. [49], in which older participants completed cognitive tests in the laboratory (face-to-face) and in the laboratory (other room) via videoconference (ICC: 0.79). The latter may explain the slightly better ICC values observed in the study of Wadsworth et al. [49] because their assessments were not conducted in a real-life setting (i.e., the participant’s home) where additional sources of bias such as Internet connection problems or fear of using digital devices without help have to be considered [49]. The indices of absolute reliability of the oral TMT B can be rated as very poor in both age groups and showed the weakest absolute reliability within our set of cognitive fitness tests. A possible explanation for the occurrence of a systematic error and the poor absolute reliability of the oral TMT B is considerable learning and practice effects because the participants performed better at the second time (i.e., remote assessment) since no alternative version of the oral TMT B is available [110]. The results of absolute reliability (SEM: ≥ 10%) suggest that the oral TMT B should not be used for remote testing via videoconference. A further issue, which has already been mentioned above and that might decrease the reproducibility of oral TMT A and B, is related to the delays in video transmission (e.g., due to an unstable internet connection) which deteriorate timing-sensitive measures in the remote condition. Thus, future studies that use videoconferencing to assess time-sensitive cognitive measures are advised to take possible issues with time delays arising from unstable internet connection into account [111].

The results of the two memory tests namely word list immediate recall and word list delayed recall point towards a moderate to good relative reliability in both cohorts. The absolute reliability for the younger adults reached an acceptable level for the word list delayed recall but a poor level for the word list immediate recall (for more details see Table 2). Conversely, in our sample of MOA, the word list delayed recall shows poor absolute reliability whereas the word list immediate recall demonstrates acceptable absolute reliability (for more details see Table 4). Thus, a clear pattern regarding reproducibility is not apparent. Despite the use of alternative test versions for the memory tests [112], these inconclusive results are probably related to the influence of several factors including but not limited to daily fluctuations and the occurrence of some practice and learning effects (familiarity effect). The latter is perhaps attributable to the relatively short test–retest interval of seven days which was not sufficient enough to diminish potential practice/learning effects. Studies examining the word list immediate recall and word list delayed recall tests in isolation are hardly available in the literature because memory tests are typically included in larger test batteries [41, 47] for whom findings of single tests are often not reported in much detail which, in turn, makes a more nuanced interpretation of our current findings on memory tests difficult.

### Reproducibility of physical fitness measurements

In the current study, we used well-established physical fitness assessments that are widely used in different settings and require a minimum of space and equipment. Although the 5-time-sit-to-stand-test is one of the most frequently used clinical tests to assess physical fitness, there is still limited evidence on the reproducibility between laboratory and remote conditions [113]. Our study at least partly fills this knowledge gap because our results provide evidence for good relative reliability of the 5-time sit-to-stand test, with acceptable absolute reliability in younger adults and MOA. The findings of our study are consistent with the observation of Peyrusqué and colleagues [36], who asked 15 older adults to complete a physical performance assessment in the laboratory and one week later at home via videoconference, and observed excellent relative reliability (ICC: 0.96) and acceptable absolute reliability (SEM: 6.5%) of the 5-time-sit-to-stand-test [36]. Furthermore, our observations fit the findings of Buckinx et al. [38], who assessed in 45 healthy older adults (77.7 ± 7.7 years) the 5-time-sit-to-stand-test performance remotely and face-to-face (both measurements at the participants’ homes), although they reported slightly better results in terms of relative reliability and absolute reliability (ICC: 0.97, SEM: 6.658%) than our study. The larger sample size and the face-to-face measurement at home, in contrast to our study in the laboratory, may explain the slightly better reproducibility in the study of Buckinx et al. [38].

Nevertheless, our results from the 5-time-sit-to-stand-test must be interpreted with some caution given that a statistically significant difference was found between the study settings in both younger adults and MOA, which points towards a systematic error. As with the time-dependent oral TMT measurement, we assume that this observation is caused by delays in digital transmission. In particular, this line of interpretation and the occurrence of such a bias is buttressed by the fact that the younger adults (lab: 7.73 s vs. remote: 8.25 s) and MOA (lab: 9.58 s vs. remote: 10.49 s) required more time to complete the 5-time-sit-to-stand-test in the remote assessment as compared to the laboratory-based assessment.

The balance test, which is based on the work of Theisen and Wydra [88], exhibited moderate relative reliability and acceptable absolute reliability in both cohorts. Thus, our findings fit the observation of Hoenemeyer et al. [34], Pelicioni et al. [37], and Peyrusqué et al. [36], who studied the reproducibility of laboratory and remotely conducted balance assessments in cancer survivors and healthy older adults [34, 36, 37]. The lack of studies that consider absolute reliability limits the comparison of absolute indices using the SEM. The studies by Bucknix et al. (SEM: 24.03%) [38] and Peyrusqué et al. (SEM: 15.5%) [36] observed a lower absolute reliability (i.e., indicated by a higher SEM) for balance measures as compared to our results (SEM: 5.7—9.5%) between the laboratory-based and remote-based balance examinations. Given that in both studies different balance tests were applied, it seems reasonable to assume that the above-reported discrepancies could be related to the utilization of different balance tests. In addition, the study of Peyrusqué et al. [36] and the study by Pelicioni et al. [37] showed good relative reliability in healthy older adults (unipedal-balance: ICC 0.79; Berg-Balance-Test ICC: 0.82). Notably, the two studies showed slightly higher ICCs in comparison to the values obtained in our study (for more details see Tables 2 and 4). Despite the fact that both studies had a relatively small sample size (15 participants) and used different balance tests, another reason for this difference could be the higher heterogeneity in various factors (e.g., age, gender, lifestyle, and education) in the samples of Peyrusqué et al. [36] and Pelicioni et al. [37].

In addition to the above-mentioned factors, technical challenges such as the low quality of the video, poor positioning of the camera, and limited space for balance assessment, may also have introduced some bias, which, in turn, lowered the reproducibility of the balance performance. The studies by Hoenemeyer et al. [34], Pelicioni et al. [37], and Peyrusqué et al. [36], as well as the review by Heslop et al. [16] exclusively focused on older participants. Thus, our findings concerning younger adults add new evidence to the literature. The relative reliability of the balance test among our sample of younger adults can be rated as moderate with an ICC of 0.54. The performance of the younger adults was, on a descriptive level, slightly lower than that of our sample of MOA (ICC: 0.67). This difference might be attributable to the fact that the balance test in our study was originally developed for older adults, and thus ceiling effects may have occurred in the younger participants. Such ceiling effects may contribute, among other factors such as the susceptibility of ICC determination to the between-subject variability [114, 115] (i.e., lower SD in younger adults), to the poorer relative reliability observed in our sample of younger adults [116]. Thus, we recommend that future research should consider the application and evaluation of device-based balance tests (e.g., via wearables) to achieve a more objective and accurate assessment of postural control (e.g. center of pressure) and to reduce potential ceiling effects [15].

In the current study, we used a step test that was based on the recommendations of Matthews et al. [39] who proposed an optimized and modified YMCA step test with different step heights for a digital implementation. They reported a good to excellent inter-day reliability with a test–retest interval of 13 ± 10 days for home-based assessments in healthy younger adults [39]. Our study extends the findings by Matthews et al. [39] because an acceptable absolute and excellent relative reliability between laboratory-based and home-based assessments for both younger adults and MOA was observed. Regarding our sample of younger adults, our findings are consistent with a previous study in healthy younger adults (27.25 ± 4 years) which examined the test–retest reliability of a home-based 3-min step test that was self-administered via the R Plus Health app [117]. In this study, a good to excellent relative and absolute reliability (ICC: 0.92, SEM: 5.18 beats per minute) was reported for a test–retest interval of two to three days [117]. Concerning older adults, our findings are comparable to the study of Hoenemeyer et al. [34] who noticed that in older cancer survivors (56.5 years) the execution of a step test (i.e., modified form with knee lifts without stepping on a box) is feasible and has good relative reliability (ICC: 0.87), and has acceptable absolute reliability when comparing laboratory-based and remote assessments [34]. In addition, Buckinx et al. [38], who asked healthy older participants (77.7 ± 7.7 years) to complete a 2-min step test remotely and face-to-face at their home, observed a slightly poorer intra-observer relative and absolute reliability (ICC = 0.85, SEM = 11.20%) as compared to our study. Similar to the study by Hoenemeyer et al. [34], Buckinx et al. [38] used a modified step test (i.e., knee lifts without stepping on a box) which may have led to slightly different results in terms of reproducibility.

Taken together, although the physical fitness assessments were somewhat more challenging in terms of digital implementation (e.g., evaluation of execution, use of the Elite HRV app), our study provides further evidence that in younger adults and MOA physical fitness tests such as the step test which assesses endurance capacity can be successfully and reliably performed via remote assessments that use videoconference software.

### Comparison between younger adults and MOA

Younger adults are generally more familiar with digital devices [118–120]. Thus, one is perhaps prone to assume that younger adults engage more easily in RFA then older ones. However, although only the MOAs reported some nervousness before the remote assessment, and the investigator required slightly more time to familiarize them with the use of Zoom® and the Elite HRV app the RFA was generally well-tolerated and accepted in this age group. Hence, some training in digital applications prior to RFA is likely to further enhance digital literacy and, in turn, to reduce barriers for utilizing RFA in older persons [121].

In addition, we found that in both younger adults and MOAs the laboratory-based and remote assessment of the step test and HR measurement were highly comparable. The latter finding suggests that the step test and HR measurement are promising candidates to be applied in future studies with remote measurements, regardless of the age of the participants. Overall, our data did not provide evidence or show a clear trend that the reproducibility of laboratory-based to remote assessments is influenced by the age of the participants. Instead, the reproducibility level strongly depended on the specific measures of physical and cognitive fitness – irrespective of age.

### Feasibility and implementation

Our results provide evidence that the assessment of selected parameters of HR(V), physical and cognitive fitness performance via videoconferencing is feasible in healthy younger adults and MOA. Some authors have requested more well-designed studies before an RFA can be unreservedly recommended [16]. The current study adds to the existing evidence [31, 34, 103], that RFA is indeed both feasible and reliable. Nevertheless, in addition to the challenges of RFA outlined by Heslop et al. [16], various difficulties and obstacles in conducting RFA utilizing digital technologies should be taken into account. In particular, factors such as inadequate lighting, limited space for a suitable camera angle, or lack of space for the execution of the (physical fitness) tests can often interfere with optimal data collection [122]. Additionally, some participants in our study reported concentration problems in their own homes, because it was not always possible to find an isolated and quiet room without distractions, whereas others experienced remote assessments in their homes as more comfortable compared to the laboratory.

For RFA via videoconference software, a stable internet connection is an important prerequisite (especially for time-sensitive tests) but does not ensure that no technical problems occur (e.g., connection errors leading to a time delay that influences time-based measures). In this context, a nationwide digital infrastructure in Germany (with digital devices in every household) is not yet standard, and unequal access to digital infrastructure in the population, also referred to as “digital divide” [123], which primarily affects older people with a low income, low education, and those living alone with chronic diseases who rarely use digital healthcare services [124], are further hurdles for the implementation of RFA in a larger scale. Thus, there is some risk that precisely those people who urgently require healthcare (e.g., older adults with chronic diseases) will not benefit from digital services, but could become as a consequence of the digital divide even more isolated [12]. Hence, increasing the accessibility and enhancing the digital skills of the population, especially the vulnerable older adults, should be given a high priority in future public health actions to unleash the full potential of digital health applications (e.g., RFA) and to improve health equity.

Nevertheless, RFA may improve the representativeness of health research because it can be used to recruit samples from diverse locations, including rural areas, which may be underrepresented otherwise [1, 4, 12]. Remote assessment can also benefit healthcare. In particular, RFA allows to deliver specific healthcare services in rural areas without the need to travel long distances or without personal contact in times of pandemic restrictions. This can alleviate the individual burden and the problem of accessibility of healthcare services in rural areas or during challenging circumstances [3, 7]. In addition, RFA may also be advantageous for preventive care and adults with chronic diseases (e.g., allowing for the early detection of deficiencies and symptoms). Such early detection can enable healthcare providers to initiate preventive interventions at an earlier stage of chronic diseases such as dementia, which might result in better treatment outcomes.

## Limitation

Some limitations in this study should be taken into account when interpreting our findings. The participants in this study were predominantly healthy adults with a high level of education and access to digital devices and the internet. In addition, all participants were recruited from Potsdam/Berlin and the surrounding area. Therefore, the findings of the study cannot be readily generalized to individuals who reside in more rural areas, those with varying levels of education, or individuals having specific diseases. Another important point to be considered is the varying level of digital literacy among the participants which was not empirically assessed but which may have influenced the results of the remote testing. Thus, further studies that address the aforementioned limitations and differentiate age groups based on narrower intervals (e.g., 10-year intervals) are required to draw more robust and nuanced conclusions.

In addition to the issue of limited generalisability, the complexity of interpreting reliability, especially absolute indices of reliability, must also be taken into account. A comparison between laboratory- and remote-based assessments depends on the sample’s characteristics, the assessed outcome parameters, and the contexts. For example, there are currently only cut-off values for the cognitive assessments available, but no more fine-graded differentiation in terms of clinically relevant differences, so that the absolute reliability (i.e., SEM) needs to be interpreted with caution. In this context, it should also be considered that measures relying on absolute difference are context-sensitive (e.g., variability in HR of 6 bpm in a sedentary sample of the general population will be interpreted differently than in a sample of physically active participants.

Concerning the HRV measurement, future studies should aim to control the menstrual cycle of the participating women because HRV can differ at different cycle phases [106]. In addition, the breathing pattern of the participants was not controlled during the resting HRV measurement (spontaneous breathing), which might influence HRV, although it has to be acknowledged that the current evidence on this issue is still inconsistent [101, 125, 126]. It is also important to note that we used a more liberal artefact correction in our pre-processing of the HRV data to ensure the representativeness of the sample (fewer exclusions). Regarding the cognitive tests, it is important to consider that the DemTect from which the word list was taken has been developed for individuals with suspected cognitive disorders, and the quality criteria were evaluated based on this group. Here, we used this cognitive test in healthy adults because it provides the advantages of being simple, brief, and auditory.

Another point that should be considered when interpreting our findings is related to the study design and includes, but is not limited to the randomisation of the study setting (i.e., laboratory and remote assessment). Comparable to other studies [34, 36], we did not conduct a randomisation of the conditions in the current study because of organisational reasons and the technical support provided by the investigator when installing the app (including test instructions). In this context, some of our participants stated that a prior face-to-face introduction of the equipment was necessary and helpful because they felt that otherwise the remote tests would have been too difficult for them. In the current study, we used a fixed order of conditions (i.e., laboratory assessments followed by remote assessments) to ensure ecological validity and mirror a real-life setting (e.g., the individual is first examined in the hospital or rehabilitation clinic which is followed by remote follow-up assessments via digital devices in the home of the participant). However, further studies investigating whether randomization influences reproducibility are needed to allow for a more nuanced interpretation of our findings. For a transfer to clinical practice, a hybrid model, as described in the article by Salvadori and Pantoni [127], might be an appropriate approach to tackle the problems mentioned above.

Moreover, it was not feasible to randomize the individual tests within the assessment sessions to avoid the potential influence of one test on another test (e.g., step test on resting-state HRV). In addition, the test–retest interval of seven days does not allow us to readily generalize our findings to longer time intervals between assessments (e.g., as typically used in longitudinal observational studies or interventional studies). The test–retest interval of seven days was chosen to account for the fact that physiological measures (i.e., HRV) probably show higher physiological fluctuations across longer test–retest intervals due to many internal and external influencing factors [74, 128]. Such higher physiological fluctuations, however, would have contradicted our study aim (e.g., to compare laboratory-based and remote assessments). However, further studies are advised to use longer test–retest intervals to gain a more comprehensive understanding of the effects of time on the reproducibility of specific measures of physical and cognitive fitness which is required to inform the conceptualization of longitudinal studies and to assist practitioners in real-world data interpretation. Finally, it should be noted that due to resource constraints, no blinding was performed in this study because the same investigator conducted the tests and statistical analyses.

## Conclusion

The current study, which investigated in younger adults and MOA the feasibility and reproducibility of RFA to laboratory-based assessments, showed that the RFA of selected measures of physical and cognitive fitness via videoconference software is feasible and that the captured data is comparable to laboratory-based assessments. The results were not influenced by the age of the participants. However, it needs to be acknowledged that the reproducibility varies depending on the specific measure of interest. Further research is required to extend the current findings to ecologically more valid settings (e.g., in terms of routine health care in rural areas) and other cohorts (e.g., people with specific diseases).

## Supplementary Information

Supplementary Material 1.

Supplementary Material 2.

## Data Availability

The raw data will be made available by the authors, upon written request.

## Abbreviations

DFAa1: Short-term scaling exponent alpha1 of detrended fluctuation analysis
HR: Heart rate
HRV: Heart rate variability
ICC: Intraclass correlation coefficient
Ln: Natural log-transformed
LoA: Limits of agreement
MOA: Middle-aged to older adults
PASQ: Physical Activity and Sports Questionnaire
PSS: Perceived Stress Scale
PSQI: Pittsburgh Sleep Quality Index
RFA: Remote fitness assessment
RMSSD: Square root of mean squared difference of successive RR intervals
SDNN: Standard deviation of normal-to-normal RR intervals
SEM: Standard error of measurement
SRSS: Brief Sport Recovery and Stress Scale
TMT: Trail Making Test
VO_2MAX_: Maximum oxygen uptake
