# Supplementary material for: Remote fitness assessment in younger and middle-aged to older adults: a comparison between laboratory- and videoconference-based assessment of selected measures of physical and cognitive fitness
Source: BMC Sports Sci Med Rehabil. 2024 Sep 25;16:198. doi: 10.1186/s13102-024-00985-4 (PMC11426110; doi:10.1186/s13102-024-00985-4)
Supplement: Supplementary file 1 — Supplementary Material 1. [file 13102_2024_985_MOESM1_ESM.docx]

**Supplementary Material to: “Remote Fitness Assessment in Younger and Middle-aged to Older Adults: A Comparison between Laboratory- and Videoconference-based Assessment of Selected Measures of Physical and Cognitive Fitness”**

Paula Theobald ^a^*, Fabian Herold ^a^, Thomas Gronwald ^b,c^, Notger G. Müller ^a^

^a^ Research Group Degenerative and Chronic Diseases, Movement, Faculty of Health Sciences Brandenburg, University of Potsdam, Potsdam 14476, Germany

_b_ Institute of Interdisciplinary Exercise Science and Sports Medicine, MSH Medical School Hamburg, Hamburg 20457, Germany

_c_ G-Lab, Faculty of Applied Sport Sciences and Personality, BSP Business and Law School, Berlin 12247, Germany

* Correspondence: Paula Theobald (paula.theobald@uni-potsdam.de)

Content

[1. Methods: 2](#_Toc162940787)

[i. Flow chart 2](#_Toc162940788)

[ii. Instruction of the balance test: 2](#_Toc162940789)

[iii. Instruction of the step test: 3](#_Toc162940790)

[2. Results: 4](#_Toc162940791)

[i. Paired t-test 4](#_Toc162940792)

[ii. ICC and SEM of all participants 6](#_Toc162940793)

[iii. LOA of all participants 6](#_Toc162940794)

[References 7](#_Toc162940795)

# Methods:

## Flow chart


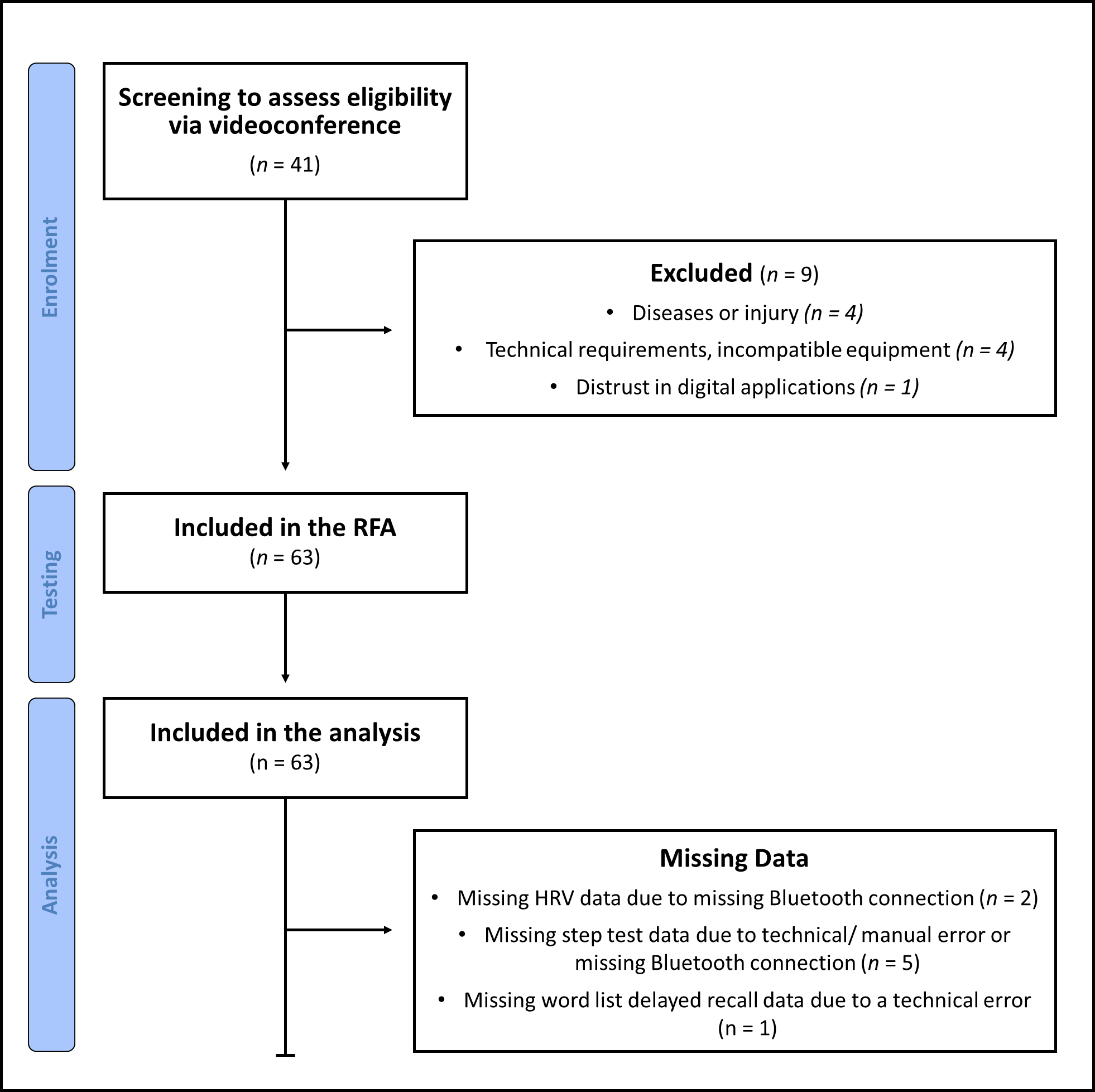


Figure S1 Flow chart of enrolment and missing data

## Instruction of the balance test:

In all tests (a.-f.), the participants were asked to stand as stable (motionless) as possible in an upright posture with their arms adducted in a neutral position at the side of their body, and with their view facing forward. The participants were asked to maintain the specific posture for at least 15 seconds (see below a.-f.). If the participants were instructed to place their feet behind each other, they were allowed to freely choose the front foot. If the participants were instructed to stand on one leg, they were asked to use the "preferred" leg and to raise the other leg in front of their body (knee and hip flexion 90°) with the toe pointing in the direction of their view (i.e., forward).

1. Position: feet closed, parallel together, eyes open
2. Position: feet closed, parallel together, eyes closed
3. Position: feet one behind the other, eyes open
4. Position: one-leg stand, eyes open
5. Position: feet one behind the other, eyes closed
6. Position: one-leg stand, eyes closed

The performance of the above-described balance task was evaluated using the specific scoring system detailed below (maximum points: 18)

*Evaluation criteria for scoring:*

*0 = position kept < 15 seconds; loss of balance/postural stability; eyes open.*

*1 = strong body sway; attempted to use arm and leg movements to keep balance/ postural stability; feet changed the position.*

*2 = slight body sway; attempted to use arm movements to keep balance/ postural stability.*

*3 = absence of body movements to keep balance/ postural stability.*

## Instruction of the step test:

In preparation for the step test, the step frequency was calculated, adapted to the height of the step box (see Figure 2) based on the following formula provided by Matthews et al. (2021): step frequency [steps/min] = (25.8 [mL/kg/min] – 3.5 [mL/kg/ min])/(0.2 + 1.33 × 1.8 × 0.27 (step height)[m]) = 26.35 [steps/min]; 26.35 × 4 = **105 bpm** (metronome setting). The VO_2_ for all step tests was set at 25.8 mL/kg/min to meet the estimated VO_2_ of the YMCA step test (VO_2_ = 25.8 mL/kg/min) [1, 2]. The participants were asked to step on a box with standardized dimensions (height: 27 cm). This standardized box provided the advantage that the participants were able to easily take them to their homes, either by train, bicycle, or on foot, and that the box was stable enough to support the participants' body weight during the step test. As the box often shifted on slippery surfaces such as parcels, we recommended that the participants use a sports mat or carpet as a base for the box when performing the step test.

The Elite HRV app (Elite HRV Inc., Asheville, NC, USA) was used to record and monitor heart rate (HR) before (30 seconds positioning), during (3 minutes exercise during the step test), and after the step test (90 seconds recovery) resulting in a total measurement time of 5 minutes. After the participants started the recording in the app, they had 30 seconds to position/prepare themselves in front of the box and listen to the beat of the metronome (the metronome was started by the investigator). After 30 seconds of positioning/preparation, the investigator gave the signal to start the step test, in which the participants stepped up and down on the box following the rhythm of the metronome (i.e., step up, step up – step down, step down). Participants were asked to start with their preferred leg but were allowed to switch their “leading” leg during the test if necessary. The investigator monitored the participants' HR via the app for the entire duration of the test and provided information on the elapsed time every 30 seconds. Once the step test (exercise) was completed, the investigator gave a stop signal, and then the participants were instructed to sit down and rest (90 seconds) until the Elite HRV App provided a sound signal indicating that the measurement was terminated. Finally, the participants were asked to save the recorded data in the Elite HRV App.

The average HR of the recovery time (1-minute post-exercise) was analysed via Kubios 3.5.0 software (Kubios, Ltd., Kuopio, Finland) and used to determine the individual maximum oxygen uptake for each participant using the following formula: VO_2MAX_ [mL/kg/min] = -0.2805 × HR (1-minute post-exercise) + 76.71 [1].


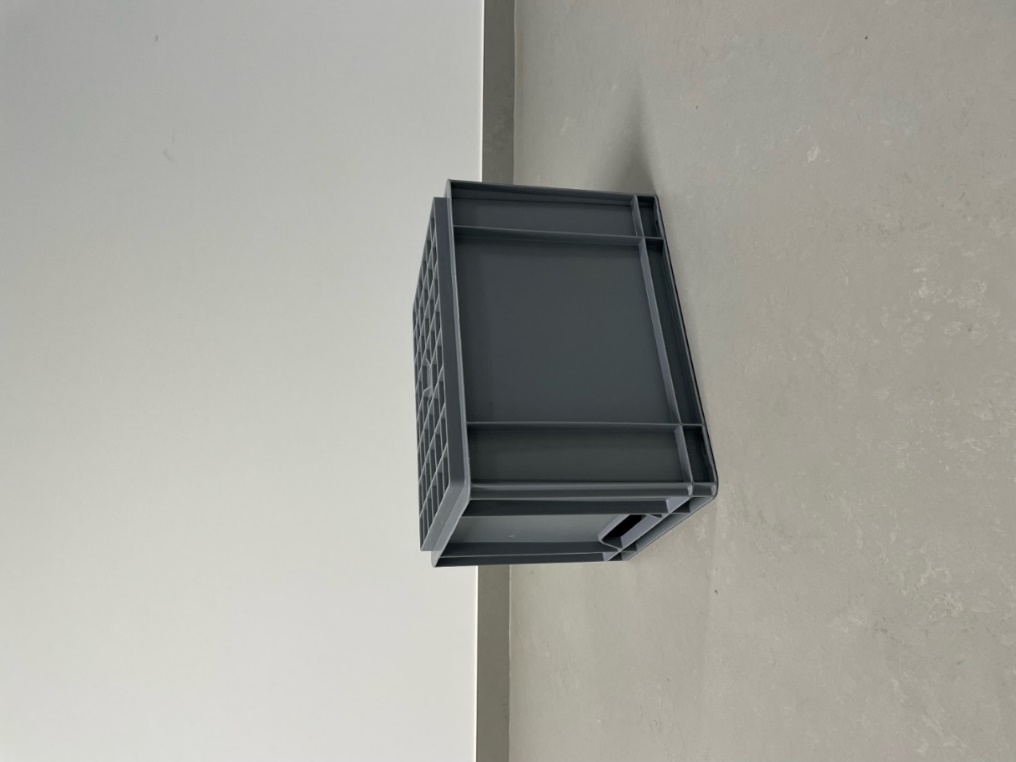


Figure S2 Picture of the step box (height: 27cm) that has been used for the step test.

# Results:

## Paired t-test

Table S1 Overview of the results of the paired t-tests comparing laboratory and remote assessment of the selected measures of physical and cognitive fitness in the cohort of **younger adults**

| Measure | t | p | Cohen’s d |
| --- | --- | --- | --- |
| lnRMSSD (ms)  [n=30] | 1.36 | 0.185 | 0.25 |
| lnSDNN (ms)  [n=30] | 1.26 | 0.218 | 0.23 |
| DFAa1  [n=30] | 0.56 | 0.580 | 0.10 |
| Resting HR (bpm)  [n=30] | 0.98 | 0.335 | 0.18 |
| Step test  (VO_2MAX_ [mL/kg/min])  [n=28] | 1.52 | 0.139 | 0.29 |
| Oral TMT A (sec)  [n=31] | 2.48 | 0.019* | 0.45 |
| Oral TMT B (sec)  [n=31] | 1.48 | 0.150 | 0.27 |
| Digit span (points)  [n=31] | 0.80 | 0.432 | 0.14 |
| Word list immediate recall (points) [n=31] | 1.23 | 0.229 | 0.22 |
| Word list delayed recall (points) [n=30] | 0.35 | 0.732 | 0.06 |
| Balance (points)  [n=31] | 1.09 | 0.284 | 0.20 |
| 5-time-sit-to-stand (s)  [n=31] | 3.18 | 0.003* | 0.57 |
| Recovery (SRSS)  [n=31] | 1.15 | 0.260 | 0.21 |
| Stress (SRSS)  [n=31] | 0.51 | 0.611 | 0.09 |
| Sleep quality (SRSS) [n=31] | 1.73 | 0.094 | 0.31 |
| Sleep duration (SRSS) [n=31] | 1.76 | 0.088 | 0.32 |

Bpm = beats per minute; DFAa1 = short-term scaling exponent alpha1 of detrended fluctuation analysis; HR = heart rate; ln = natural log-transformed; RMSSD= square root of mean squared difference of successive RR intervals; SDNN = standard deviation of normal-to-normal RR intervals; SRSS = Brief Sport Recovery and Stress Scale; TMT = Trail Making Test; VO_2MAX_ = maximum oxygen uptake; * = significant result (p < 0.05). Please note Cohen’s d was rated as follows: small (d ≥ 0.2), moderate (d ≥ 0.5), and large (d > 0.8) [3].

Table S2 Overview of the results of the paired t-tests comparing the laboratory and remote assessments of the selected measures of physical and cognitive fitness in the cohort of **middle-aged to** **older adults (MOA)**

| Measure | t | p | Cohen’s d |
| --- | --- | --- | --- |
| lnRMSSD (ms)  [n=31] | 0.05 | 0.962 | 0.01 |
| lnSDNN (ms)  [n=31] | 0.38 | 0.709 | 0.07 |
| DFAa1  [n=31] | 0.76 | 0.451 | 0.14 |
| Resting HR (bpm)  [n=31] | 0.99 | 0.164 | 0.18 |
| Step test  (VO_2MAX_ [mL/kg/min])  [n=30] | 1.39 | 0.175 | 0.25 |
| Oral TMT A (sec)  [n=32] | 3.16 | < 0.001* | 0.66 |
| Oral TMT B (sec)  [n=32] | 0.55 | 0.585 | 0.10 |
| Digit span (points)  [n=32] | 0.93 | 0.361 | 0.16 |
| Word list immediate recall (points) [n=32] | 0.08 | 0.937 | 0.01 |
| Word list delayed recall (points) [n=32] | 0.48 | 0.635 | 0.09 |
| Balance (points)  [n=32] | < 0.00 | 1.000 | 0.00 |
| 5-time-sit-to-stand (s)  [n=32] | 2.92 | 0.006* | 0.50 |
| Recovery (SRSS)  [n=32] | 0.70 | 0.489 | 0.12 |
| Stress (SRSS)  [n=32] | 1.50 | 0.143 | 0.27 |
| Sleep quality (SRSS)  [n=31] | 0.38 | 0.708 | 0.07 |
| Sleep duration (SRSS) [n=31] | 0.75 | 0.462 | 0.13 |

Bpm = beats per minute; DFAa1 = short-term scaling exponent alpha1 of detrended fluctuation analysis; HR = heart rate; ln = natural log-transformed; RMSSD= square root of mean squared difference of successive RR intervals; SDNN= standard deviation of normal-to-normal RR intervals; TMT = Trail Making Test; VO_2MAX_ = maximum oxygen uptake; * = significant result (p < 0.05). Please note Cohen’s d was rated as follows: small (d ≥ 0.2), moderate (d ≥ 0.5), and large (d > 0.8) [3].

## ICC and SEM of all participants

Table S3 Overview of the intraclass correlation coefficient (ICC) and the standard error of measurement (SEM) for the comparison between laboratory and remotely assessed measures of physical and cognitive fitness using the data of **all participants** (i.e., pooled data of younger and MOA).

| Measure | Lab  Mean (SD) | Remote  Mean (SD) | ICC (95% CI) | p-value | SEM (%)^a^ |
| --- | --- | --- | --- | --- | --- |
| lnRMSSD (ms)  [n=61] | 3.39 (0.62) | 3.45 (0.68) | 0.73 (0.58-0.83) | <0.001 | 0.34 (10.0%) |
| lnSDNN (ms)  [n=61] | 3.57 (0.56) | 3.60 (0.59) | 0.776 (0.65-0.86) | <0.001 | 0.27 (7.6%) |
| DFAa1  [n=61] | 1.12 (0.30) | 1.13 (0.31) | 0.582 (0.39-0.73) | <0.001 | 0.20 (17.6%) |
| Resting HR (bpm)  [n=61] | 71.16 (12.68) | 71.09 (11.75) | 0.81 (0.69-0.88) | <0.001 | 0.30 (7.5%) |
| Step test  (VO_2MAX_ [mL/kg/min])  [n=58] | 44.55 (5.92) | 45.10 (5.83) | 0.94 (0.89-0.96) | <0.001 | 1.47 (3.3%) |
| Oral TMT A (sec)  [n=63] | 9.84 (3.57) | 10.85 (4.13) | 0.86 (0.70-0.93) | <0.001 | 1.44 (13.9%) |
| Oral TMT B (sec)  [n=63] | 31.66 (15.86) | 29.37 (14.87) | 0.68 (0.52-0.79) | <0.001 | 8.69 (28.5%) |
| Digit span (points)  [n=63] | 19.27 (3.747) | 18.90 (3.86) | 0.79 (0.68-0.87) | <0.001 | 2.09 (11.0%) |
| Word list immediate recall (points)  [n=63] | 15.08 (2.68) | 15.35 (2.52) | 0.57 (0.37-0.71) | <0.001 | 1.71 (11.2%) |
| Word list delayed recall (points) [n=62] | 7.03 (2.02) | 6.92 (1.86) | 0.70 (0.54-0.81) | <0.001 | 1.06 (15.2%) |
| Balance (points)  [n=63] | 14.71 (2.59) | 14.59 (2.10) | 0.779 (0.66-0.86) | <0.001 | 1.10 (7.5%) |
| 5-time-sit-to-stand (s)  [n=63] | 8.67 (2.49) | 9.38 (3.03) | 0.85 (0.70-0.92) | <0.001 | 1.09 (12.1%) |

Bpm = beats per minute; CI = Confidence interval; DFAa1 = short-term scaling exponent alpha1 of detrended fluctuation analysis; ICC = Intra-class correlation coefficient; ln = natural log-transformed; RMSSD= square root of mean squared difference of successive RR intervals; SDNN= standard deviation of normal-to-normal RR intervals; SD = standard deviation; TMT = Trail Making Test; VO_2MAX_ = maximum oxygen uptake; ^a^ = SEM expressed as a percentage of the mean laboratory and remote score.

Please note the ICC was rated as follows: poor (< 0.5), moderate (0.5- 0.75), good (0.75 -0.9), and excellent (≥ 0.9) [4]. In addition, we consider an SEM smaller than 10% as acceptable [5–7].

## LOA of all participants

Table S4 Overview on the Limits of Agreement (LoA) for the comparison between the laboratory and remotely assessed measures of physical and cognitive fitness using the data of **all participants** (i.e., pooled data of younger and middle-aged to older adults).

|  |  | Limits of Agreement | |
| --- | --- | --- | --- |
| Measure | Mean (SD) Difference (Laboratory -Remote) | Lower Limit | Upper Limit |
| lnRMSSD (ms)  [n=61] | -0.06 (0.48) | -1.01 | 0.89 |
| lnSDNN (ms)  [n=61] | -0.03 (28.07) | -0.79 | 0.73 |
| DFAa1  [n=61] | -0.01 (0.28) | -0.56 | 0.55 |
| Resting HR (bpm)  [n=61] | 0.07 (7.68) | -14.99 | 15.12 |
| Step test  (VO_2MAX_ [mL/kg/min])  [n=58] | -0.56 (2.05) | -4.58 | 3.47 |
| Oral TMT A (sec)  [n=63] | -1.00 (1.80) | -4.54 | 2.52 |
| Oral TMT B (sec)  [n=63] | 2.29 (12.13) | -21.65 | 26.24 |
| Digit span (points)  [n=63] | 0.36 (2.44) | -4.43 | 5.16 |
| Word list immediate recall (points) [n=63] | -0.27 (2.42) | -5.01 | 4.47 |
| Word list delayed recall (points) [n=62] | 0.11 (1.52) | -2.87 | 3.09 |
| Balance (points)  [n=63] | 0.13 (1.57) | -2.95 | 3.20 |
| 5-time-sit-to-stand (sec)  [n=63] | -0.71 (1.40) | -3.46 | 2.03 |

DFAa1 = short-term scaling exponent alpha1 of detrended fluctuation analysis; HR = heart rate; ln = natural log-transformed; RMSSD = square root of mean squared difference of successive RR intervals; SDNN = standard deviation of normal-to-normal RR intervals; SD = standard deviation; TMT = Trail Making Test; VO_2MAX_ = maximum oxygen uptake.

References

1. Matthews EL, Horvat FM, Phillips DA. Variable Height Step Test Provides Reliable Heart Rate Values During Virtual Cardiorespiratory Fitness Testing. Measurement in Physical Education and Exercise Science. 2021:1–10. doi:10.1080/1091367X.2021.1964507.

2. Liguori G, Feito Y, Fountaine CJ, Roy B, editors. ACSM's guidelines for exercise testing and prescription. Philadelphia, Baltimore, New York, London, Hong Kong, Sydney, Tokyo: Wolters Kluwer; 2022.

3. Cohen J. Statistical Power Analysis for the Behavioral Sciences. 2nd ed. Hoboken: Taylor and Francis; 2013.

4. Koo TK, Li MY. A Guideline of Selecting and Reporting Intraclass Correlation Coefficients for Reliability Research. J Chiropr Med. 2016;15:155–63. doi:10.1016/j.jcm.2016.02.012.

5. Peyrusqué E, Granet J, Pageaux B, Buckinx F, Aubertin-Leheudre M. Assessing Physical Performance in Older Adults during Isolation or Lockdown Periods: Web-Based Video Conferencing as a Solution. J Nutr Health Aging. 2022;26:52–6. doi:10.1007/s12603-021-1699-y.

6. Buckinx F, Rezoulat M, Lefranc C, Reginster J-Y, Bruyere O. Comparing remote and face-to-face assessments of physical performance in older adults: A reliability study. Geriatr Nurs. 2023;55:71–8. doi:10.1016/j.gerinurse.2023.11.004.

7. Weir JP. Quantifying test-retest reliability using the intraclass correlation coefficient and the SEM. J Strength Cond Res. 2005;19:231–40. doi:10.1519/15184.1.
