# Supplementary material for: Remote fitness assessment in younger and middle-aged to older adults: a comparison between laboratory- and videoconference-based assessment of selected measures of physical and cognitive fitness
Source: BMC Sports Sci Med Rehabil. 2024 Sep 25;16:198. doi: 10.1186/s13102-024-00985-4 (PMC11426110; doi:10.1186/s13102-024-00985-4)
Supplement: Supplementary file 2 — Supplementary Material 2. [file 13102_2024_985_MOESM2_ESM.docx]

**Supplementary Material: Questionnaires**

ID: Date: m

**Feasibility of remote assessments I**

Please answer the following questions as honestly and spontaneously as possible. Your answers will help us analyse the feasibility of this new approach of fitness assessment.

1. I felt comfortable during the tests in the laboratory.

| - Strongly agree | - Agree | - Neither agree or disagree | - Disagree | - Strongly Disagree |
| --- | --- | --- | --- | --- |

1. The tests in the laboratory were very difficult for me.

| - Strongly agree | - Agree | - Neither agree or disagree | - Disagree | - Strongly Disagree |
| --- | --- | --- | --- | --- |

1. The tests in the laboratory were stressful for me.

| - Strongly agree | - Agree | - Neither agree or disagree | - Disagree | - Strongly Disagree |
| --- | --- | --- | --- | --- |

1. I find it easy to carry out the tests in the laboratory.

| - Strongly agree | - Agree | - Neither agree or disagree | - Disagree | - Strongly Disagree |
| --- | --- | --- | --- | --- |

ID: Date: m

**Feasibility of remote assessments II**

Please answer the following questions as honestly and spontaneously as possible. Your answers will help us analyse the feasibility of this new method.

1. I felt comfortable during the tests at home.

| - Strongly agree | - Agree | - Neither agree or disagree | - Disagree | - Strongly Disagree |
| --- | --- | --- | --- | --- |

1. The tests at home were very difficult for me.

| - Strongly agree | - Agree | - Neither agree or disagree | - Disagree | - Strongly Disagree |
| --- | --- | --- | --- | --- |

1. The tests at home were stressful for me.

| - Strongly agree | - Agree | - Neither agree or disagree | - Disagree | - Strongly Disagree |
| --- | --- | --- | --- | --- |

1. I find it easy to carry out the tests via videoconference.

| - Strongly agree | - Agree | - Neither agree or disagree | - Disagree | - Strongly Disagree |
| --- | --- | --- | --- | --- |

ID: Date: m

**Data Sheet for Participants (Questionnaire III)**

Gender: □ female □male □diverse

Age: …………………years

1. 1. Do you have a **smartphone**?

□ yes □ no

1. Would you be willing to download an app to your **smartphone** for testing?

□ yes □ no

1. Do you have a **stable internet** connection at home?

□ yes □ no

**Medication (Please mark with a cross where applicable):**

| Antihypertensives | ⃝ |  |
| --- | --- | --- |
| Beta-blockers | ⃝ |  |
| ACE inhibitors | ⃝ |  |
| Blood thinners | ⃝ |  |
| Cardiac glycosides | ⃝ |  |
| Calcium antagonists | ⃝ |  |
| Renin inhibitor | ⃝ |  |
| Antidepressants | ⃝ |  |
| Antipsychotics | ⃝ |  |
| Antidementivum | ⃝ |  |
| Hypnotics (sleeping pills) | ⃝ |  |
| Birth control pill (oral contraception) | ⃝ |  |
| Viagra | ⃝ |  |
| Other medications | ⃝ | *• ………………………………..*  *• …………………………………* |

**Diseases (Please mark with a cross where applicable):**

|  | *Yes, medically diagnosed* | *non-existent* | *Unknown to date* | *Notes* |
| --- | --- | --- | --- | --- |
| Visual disorders | ⃝ | ⃝ | ⃝ | …………………………… |
| Hearing disorder | ⃝ | ⃝ | ⃝ | …………………………… |
| High blood pressure | ⃝ | ⃝ | ⃝ |  |
| Low blood pressure | ⃝ | ⃝ | ⃝ |  |
| Heart attack | ⃝ | ⃝ | ⃝ |  |
| Cardiac arrhythmia | ⃝ | ⃝ | ⃝ |  |
| Coronary heart disease (stenosis) | ⃝ | ⃝ | ⃝ |  |
| Angina pectoris | ⃝ | ⃝ | ⃝ |  |
| Heart valve defect | ⃝ | ⃝ | ⃝ |  |
| Heart failure/cardiac insufficiency | ⃝ | ⃝ | ⃝ |  |
| Cardiomyopathy | ⃝ | ⃝ | ⃝ |  |
| Functional heart problems | ⃝ | ⃝ | ⃝ |  |
| Stroke | ⃝ | ⃝ | ⃝ |  |
| Renal insufficiency | ⃝ | ⃝ | ⃝ |  |
| Type 1 diabetes | ⃝ | ⃝ | ⃝ |  |
| Type 2 diabetes (acquired) | ⃝ | ⃝ | ⃝ |  |
| Depressionen | ⃝ | ⃝ | ⃝ |  |
| Anxiety disorders | ⃝ | ⃝ | ⃝ |  |
| Alzheimer's dementia | ⃝ | ⃝ | ⃝ |  |
| Cognitive impairment (MCI) | ⃝ | ⃝ | ⃝ |  |
| Alcohol addiction | ⃝ | ⃝ | ⃝ |  |
| Drug addiction | ⃝ | ⃝ | ⃝ |  |
| Eating disorder | ⃝ | ⃝ | ⃝ |  |
| Migraine | ⃝ | ⃝ | ⃝ |  |
| Chronic headaches | ⃝ | ⃝ | ⃝ |  |
| Arteriosclerosis | ⃝ | ⃝ | ⃝ |  |
| Asthma | ⃝ | ⃝ | ⃝ |  |
| Autism | ⃝ | ⃝ | ⃝ |  |
| Mental disability | ⃝ | ⃝ | ⃝ |  |
| Other diseases | ⃝ | ⃝ | ⃝ | ……………………………... |
| •………………………….. | ⃝ | ⃝ | ⃝ | ……………………………... |

**Are you/can you be pregnant?**

□ yes □ no

**Do you smoke?**

⃝ No

⃝ Yes *If yes*: ⃝ less than 5 cigarettes per day

⃝ 5 to 10 cigarettes per day

⃝ 10 to 20 cigarettes per day

⃝ more than 20 cigarettes per day

***Mark the relevant statements with a cross.***

| **Alcohol consumption:**  ⃝ Never (absolute renunciation)  ⃝ Hardly (less than 1 time per month)  ⃝ Rarely (approx. 1-2 times a month)  ⃝ Occasionally (more than 2 times a month)  ⃝ Regularly (several times a week)  ⃝ Daily (more than 200ml per day) | **Drug use (illegal intoxicants):**  ⃝ Never (absolute renunciation)  ⃝ Hardly (less than 1 time per month)  ⃝ Rarely (approx. 1-2 times a month)  ⃝ Occasionally (more than 2 times a month)  ⃝ Regularly (several times a week)  ⃝ In the past (> 6 months) |
| --- | --- |

**Dietary habits (Please mark with a cross where applicable - multiple responses possible):**

| Vegan (no animal products) | ⃝ |  |  |
| --- | --- | --- | --- |
| Vegetarian (no meat and no fish) | ⃝ |  |  |
| Pescetarian (no meat, but fish) | ⃝ |  |  |
| Lactose intolerance | ⃝ |  |  |
| Other food intolerances: | ⃝ | *•* …………………………………………... |  |
| No restriction (including meat consumption) | ⃝ |  |  |
| Balanced, healthy diet | ⃝ |  |  |
| Convenience food (including fast food) | ⃝ |  |  |
| Food supplements: | ⃝ | *•* …………………………………………... |  |

***Please mark the appropriate statements with a cross.***

**What is your highest school graduation?**

⃝ No school graduation

⃝ Lower secondary school graduation

⃝ Secondary school graduation

⃝ Advanced technical school graduation

⃝ High school

⃝ Others: …………………………………………………………………………………

**Which vocational qualification do you have?**

⃝ Still in education/training

⃝ No professional qualification and no vocational training

⃝ Vocational training

⃝ University of Applied Sciences degree

⃝ University degree

⃝ Other professional qualifications:..………………………………………………………………….
